# In vitro to in vivo extrapolation and high-content imaging for simultaneous characterization of chemically induced liver steatosis and markers of hepatotoxicity

**DOI:** 10.1007/s00204-023-03490-8

**Published:** 2023-04-12

**Authors:** Fabrice A. Müller, Marianna Stamou, Felix H. Englert, Ole Frenzel, Sabine Diedrich, Laura Suter-Dick, John F. Wambaugh, Shana J. Sturla

**Affiliations:** 1 Department of Health Sciences and Technology, ETH Zurich, Schmelzbergstrasse 9, 8092 Zurich, Switzerland; 2 Center for Computational Toxicology and Exposure, Office of Research and Development, United States Environmental Protection Agency, Research Triangle Park, Durham, NC 27711 USA; 3 School of Life Sciences, University of Applied Sciences and Arts Northwestern Switzerland, 4132 Muttenz, Switzerland; 4 Swiss Centre for Applied Human Toxicology (SCAHT), 4001 Basel, Switzerland

**Keywords:** New approach method, High-content imaging, Hepatotoxicity, In vitro to in vivo extrapolation

## Abstract

Chemically induced steatosis is characterized by lipid accumulation associated with mitochondrial dysfunction, oxidative stress and nucleus distortion. New approach methods integrating in vitro and in silico models are needed to identify chemicals that may induce these cellular events as potential risk factors for steatosis and associated hepatotoxicity. In this study we used high-content imaging for the simultaneous quantification of four cellular markers as sentinels for hepatotoxicity and steatosis in chemically exposed human liver cells in vitro. Furthermore, we evaluated the results with a computational model for the extrapolation of human oral equivalent doses (OED). First, we tested 16 reference chemicals with known capacities to induce cellular alterations in nuclear morphology, lipid accumulation, mitochondrial membrane potential and oxidative stress. Then, using physiologically based pharmacokinetic modeling and reverse dosimetry, OEDs were extrapolated from data of any stimulated individual sentinel response. The extrapolated OEDs were confirmed to be within biologically relevant exposure ranges for the reference chemicals. Next, we tested 14 chemicals found in food, selected from thousands of putative chemicals on the basis of structure-based prediction for nuclear receptor activation. Amongst these, orotic acid had an extrapolated OED overlapping with realistic exposure ranges. Thus, we were able to characterize known steatosis-inducing chemicals as well as data-scarce food-related chemicals, amongst which we confirmed orotic acid to induce hepatotoxicity. This strategy addresses needs of next generation risk assessment and can be used as a first chemical prioritization hazard screening step in a tiered approach to identify chemical risk factors for steatosis and hepatotoxicity-associated events.

**Supplementary Information:**

The online version contains supplementary material available at 10.1007/s00204-023-03490-8.

## Introduction

Steatosis is a hepatic accumulation of fatty acids in greater than 5% of hepatocytes (Chalasani et al. 2012; Musso et al. 2016). This cellular phenotype is observed in nonalcoholic fatty liver disease (NAFLD), a complex spectrum of diseases that affects around 25% of the adult population (Chalasani et al. 2012; Younossi et al. 2018) and ranges from benign liver steatosis to nonalcoholic steatohepatitis (NASH), leading to cirrhosis and eventually hepatocellular carcinoma. In addition to metabolic disorders (obesity, type 2 diabetes) and genetic factors, environmental exposure to chemicals can lead to hepatic steatosis (Kaiser et al. 2012; Wahlang et al. 2013). Also, drugs, such as amiodarone, valproic acid, and cancer chemotherapeutics can cause steatosis in some patients, particularly after chronic therapy (Begriche et al. 2011; Kaiser et al. 2012; Wahlang et al. 2013; Jennings et al. 2014; Willebrords et al. 2015; Schumacher and Guo 2015). Chemically induced liver steatosis appears to induce fatty acid synthesis, decrease fatty acid β-oxidation, decrease lipoprotein export and increase uptake of free fatty acids (Pavlik et al. 2019). Yet, data derived from animal models are poorly translated to humans due to interspecies differences concerning molecular mechanism of steatosis (Soret et al. 2020).

The adverse outcome pathway (AOP) for chemically induced liver steatosis involves nuclear receptor binding, microsomal triglyceride transfer protein (MTP) or carnitine palmitoyltransferase I (CPT-1) inhibition, coenzyme A binding and carnitine depletion as the molecular initiating events that lead to triglyceride accumulation (Allen et al. 2014; Vinken 2015). Activation of several nuclear receptors, such as ER, LXR, PXR or FXR, can lead to mitochondrial dysfunction via inhibition of β-oxidation and an increase of de novo fatty acid synthesis, creating an imbalance in fatty acid metabolism leading to the accumulation of liver triglycerides and eventually steatosis. Moreover, disruption of β-oxidation induces increased production of reactive oxygen species leading to oxidative stress (Masarone et al. 2018). Furthermore, ongoing accumulation of liver triglycerides can distort the nucleus, exacerbate mitochondrial dysfunction, oxidative stress and cause endoplasmic reticulum stress. (Moya et al. 2010; Mellor et al. 2015). Chemicals that give rise to fatty liver disease may invoke several of these cellular events as a basis for identification.

There are several recent studies concerning chemically induced steatosis in vitro (Anthérieu et al. 2011, 2012; Rogue et al. 2014; Klein et al. 2016; Tolosa et al. 2016; Angrish et al. 2017; Cuykx et al. 2018; Luckert et al. 2018; Bucher et al. 2018; Allard et al. 2020; Lichtenstein et al. 2020; Lasch et al. 2021). These include at least four studies in which key events along the liver steatosis AOP were quantified in vitro (Supplementary table 1) (Donato et al. 2012; Tolosa et al. 2016; Luckert et al. 2018; Shah et al. 2021). Most recently, Shah et al. quantified mitochondrial function, lipid accumulation, endoplasmic reticulum stress, lysosomal mass, DNA texture, nuclear size, apoptosis, and cell number using a high-content imaging approach with rat hepatocytes and 51 hepatotoxicants. Moreover, these in vitro data were extrapolated to in vivo administered equivalent doses and compared to in vivo (rat) data (Shah et al. 2021). Relating in vitro bioactivity data to exposure values by means of physiologically based pharmacokinetic modeling (PBPK) and reverse dosimetry is critical in order that in vitro bioassays can be accepted for chemical hazard and eventually risk assessment by regulatory agencies (Wetmore 2015; Hartung 2018). To our knowledge, there are as yet no reports of combining an in vitro approach for simultaneous quantification of fat accumulation with other hepatotoxicity-associated relevant events in human cells with extrapolation to relevant human exposures.

In this study, we used high-content imaging to simultaneously quantify lipid accumulation, as a marker of steatosis, along with several measures of hepatotoxicity that are associated with chemically induced liver steatosis. These include mitochondrial membrane potential disruption, oxidative stress, and nuclear morphology changes. Furthermore, these were evaluated on single cell and population levels. Sixteen reference chemicals known to induce steatosis by diverse mechanisms were tested in HepaRG cells. To evaluate the physiological relevance of the resulting dose–response relationships, we integrated the in vitro data for the reference compounds with physiologically based pharmacokinetic modeling (PBPK) and reverse dosimetry to derive human dose equivalents. These levels were then compared to in vivo exposure values. Finally, on the basis of predicted nuclear receptor agonist activity, we curated and tested a panel of food-related compounds and pesticides for their potential to induce steatosis and associated hepatotoxicity.

## Materials and methods

### Chemicals and reagents

Chemicals (Supplementary table 2) were purchased from Sigma-Aldrich (St. Louis, Missouri). Stock solutions (200x) were prepared in dimethylsulfoxide (DMSO). Chemicals soluble in water were directly dissolved in cell culture medium. A mixture of oleic acid/palmitic acid (OA/PA) was combined with bovine serum albumin (BSA) in a ratio of 5.5:1 (OA/PA: BSA). OA/PA was first dissolved in DMSO and then added to treatment medium supplemented with the corresponding BSA concentration. The OA/PA medium mix was then warmed at 60 °C for 1 h (to aid dissolution), allowed to return to room temperature, and subsequently used for experiments.

### Cell culture and chemical exposure

Undifferentiated HepaRG cells were purchased from Biopredic international (Saint Grégoire, France). HepaRG cells have unlimited growth capacity and phenotypic stability, stable enzymatic activities over 4 weeks of cultivation, and similar metabolic function to primary human hepatocytes, and also avoid limitations of primary cells such as donor variability and phenotypic instability (Aninat et al. 2006; Guillouzo et al. 2007; Lambert et al. 2009; Lübberstedt et al. 2011; Andersson et al. 2012). The line contains two cell types, hepatocyte-like and cholangiocyte-like cells (Parent et al. 2004), which allows for elucidation of cell-specific toxicity (Jossé et al. 2008). Cells were seeded at a density of approximately 27′000 cells/cm^2^ into a 150 × 25 mm petri dish and were cultured for 2 weeks in William’s E medium (cat. nr.: 32,551,020, ThermoFisher Scientific, Massachusetts, USA) supplemented with 2 mM glutamine, 10% (v/v) fetal bovine serum (FBS) good forte (cat. nr.: P40-47,500, PAN-Biotech, Aidenbach, Germany), 100 U/ml penicillin and 100 µg/ml streptomycin (cat. nr.: 15,140,122, ThermoFisher Scientific, Massachusetts, USA), 5 µg/ml Gibco recombinant AOF insulin (cat. nr.: A11382II, ThermoFisher Scientific, Massachusetts, USA) and 50 µM hydrocortisone hemisuccinate (cat. nr.: H4881, Sigma-Aldrich, St. Louis, USA) at 37 °C and 5% CO_2_. Differentiation was induced by culturing the cells for another 2 weeks in the above mentioned medium supplemented with 1.7% DMSO. After 4 weeks in culture, differentiated HepaRG cells were seeded at a high density of approximately 227′000 cells/cm^2^ (72,000 cells/well with 100 µl per well) into black 96-well plates with a clear bottom (cat.nr.: 3603, Corning, New York, USA) and maintained for 10 days. After 10 days, cells were first adapted to treatment medium (culture medium containing 0.5% DMSO and 2% FBS) for 48 h and then used for cell viability and high-content imaging analysis.

### Cell viability assessment

Cell viability was analyzed using the WST-1 assay which is based on the measurement of mitochondrial dehydrogenase activity (cat. nr: ab155902, Abcam, Cambridge, UK). HepaRG cells were exposed to chemicals at defined concentrations for 24 h. Triton X-100 (0.01%) was a positive control. One hour before the end of incubation period, 10 µl of WST-1 reagent was added to each well, quickly mixed by shaking the plate, and returned to the cell culture incubator (37 °C, 5% CO_2_). After 1 h, absorbance was measured (450 nm, reference wavelength 620 nm) using a plate reader (Infinite M200 Pro, Tecan group, Männerdorf, Switzerland). Absorbance was then subtracted from the reference wavelength, corrected for background by subtracting values measured without cells, and then normalized to the solvent control, which was set to 100%. Dose–response data were fitted using drc package (Version 3.0.1) (Ritz et al. 2015) in R 3.6.1. Data were from at least 3 independent experiments with 6 technical replicates per condition.

### High-content imaging assay

**Fig. 1:**
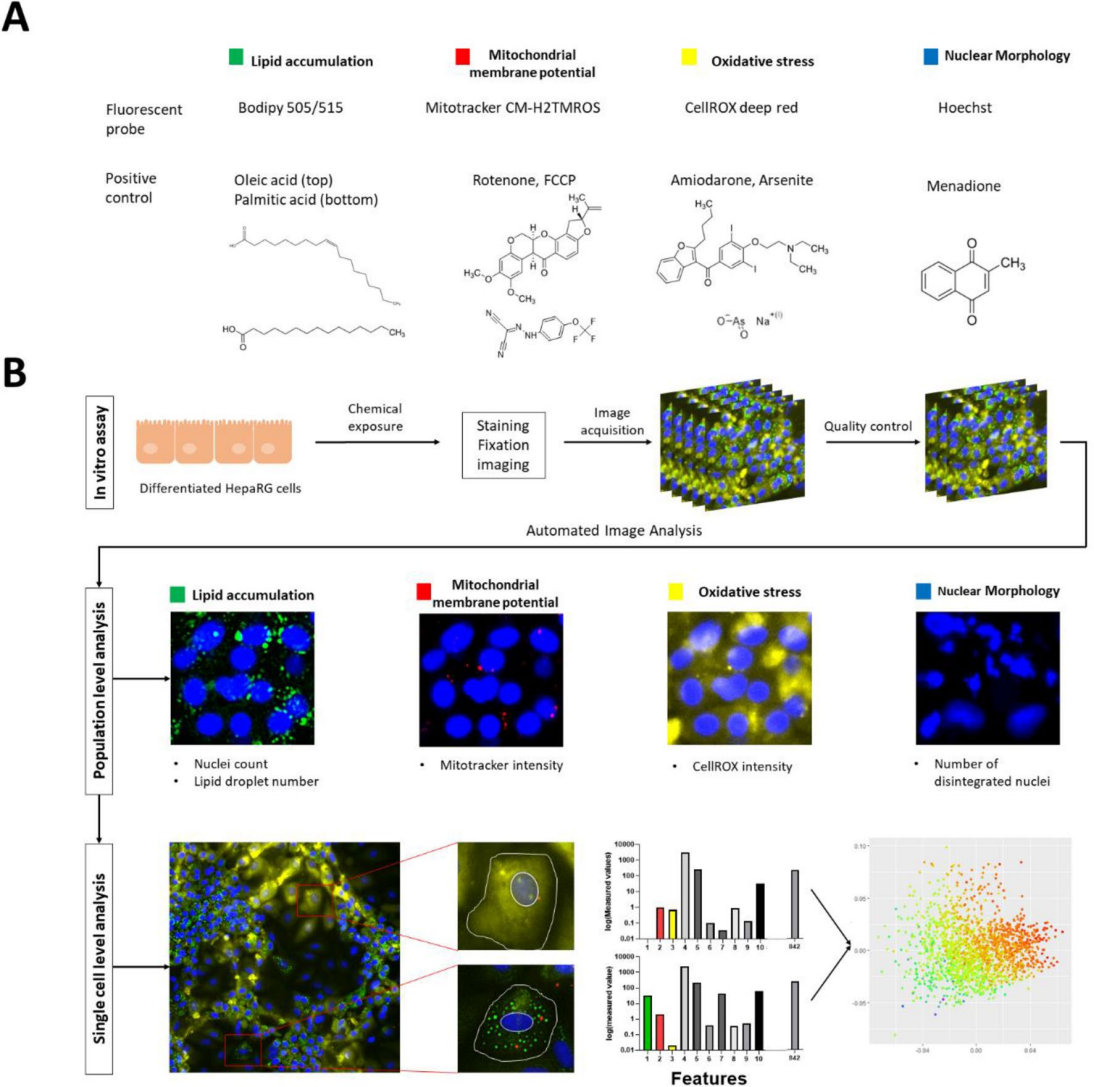
Basis of high-content imaging assay. **A** Fluorescent probes for the selected endpoints including corresponding positive controls. **B** Description of the workflow including rapid population-based quantification and detailed single cell analysis. During single cell level analysis, over 800 different features (intensity, size/shape, and texture) per cell were quantified and analyzed using principal component analysis (PCA). Plotted data were then color labeled using the values from following parameters: lipid droplet size, mitotracker intensity and CellROX intensity

Cellular responses were quantified using a high-content imaging approach (Fig. 1). Based on cell viability assessment, concentrations below the EC_50_ level were tested in the high-content imaging assay. After 24 h chemical exposure, live cells were incubated with CellROX, which reflects hydroxyl radical and superoxide anion levels (ThermoFisher Scientific 2011) (5 µM, cat. nr.: C10422, ThermoFisher Scientific, Massachusetts, USA) by adding 10 µl of a 55 µM CellROX solution in treatment medium directly to the well. After incubation (30 min, 37 °C, 5% CO_2)_, cells were rinsed twice with treatment medium without serum. After removal of the treatment medium, cells were incubated with Mitotracker Orange CM-H2TMROS which was used to measure changes in mitochondrial membrane potential (50 nM, 10 min, 37 °C, 5% CO_2_ cat. nr.: M7511, ThermoFisher Scientific, Massachusetts, USA). Subsequently, cells were washed three times with PBS and fixed with 4% PFA at room temperature for 20 min. Both dyes were retained after aldehyde fixation. After washing 3 × 5 min, cells were stained with Bodipy (50 nM, cat. nr.: D3921, ThermoFisher Scientific, Massachusetts, USA) and Hoechst (2 µM, room temperature, 15 min, cat. nr.: 62,249, ThermoFisher Scientific, Massachusetts, USA), rinsed once with PBS (cat. nr.: 14,190,250, ThermoFisher Scientific, Massachusetts, USA), and then kept in PBS. Images were acquired using an ImageXpress micro (IXM) High-Content Imaging System from Molecular Devices with a 20 × 0.75 NA S Fluor objective. A photometrics CoolSNAP HQ high resolution camera designed for quantitative fluorescence microscopy applications was set to 16 bits with a binning of one and a pixel size of 6.45 µm × 6.45 µm, and 25 images were captured per well.

### Automated quantitative image analysis

Images were quantified using the open-source software CellProfiler 3.1.8 (McQuin et al. 2018) and ImageJ 1.52 g (Schindelin et al. 2012). The image analysis pipelines are available as supplementary materials and are briefly described here. For cell population-level data, nuclei were segmented using Otsu thresholding (Sankur 2004) and everything outside the diameter range of 10–100 pixel was discarded. For lipid droplet quantification, GFP images were first deconvoluted using ImageJ 1.52 g and then imported into the CellProfiler pipeline. A binary mask of the lipid droplets then was created, segmented, and quantified using the standard settings in the “IdentifyPrimaryObjects” module (Supplementary Fig. 1). Next, mitotracker and CellROX image intensity were measured. All parameters were exported to Excel spreadsheets and analyzed in R 3.6.1, as further explained in the paragraph “Dose response data and model fitting” below. For the single cell analysis pipeline, nuclei images were used to identify individual cells. Cell borders were determined based on the mitotracker staining with the propagation method (Jones et al. 2005). All quantifications from the cell population-level pipelines were also performed at the single cell level. In addition, the following parameters were quantified per cell: size and shape of identified objects, object intensity, and object textures. The data were then exported to an SQLite database and analyzed using R 3.6.1 or GraphPad Prism 9. These pipelines can be used for a rapid population-based quantification which is amenable for high-throughput assay or for a detailed single cell analysis to identify heterogenous responses and distinct cellular phenotypes.

### Data analysis of single cell data

The single cell SQLite database file was read using RSQLite package (Müller et al. 2020). Data across three independent experiments with 6 technical replicates per concentration were summarized in a data matrix. This data matrix consisted of over 10^6^ cells with over 800 features per cell. It was imported into R 3.6.1. Constant and NA values were removed from the data matrix to perform a principle component analysis. Subsequently, the parameter cytoplasm area size was used to separate hepatocyte-like cells (< 5000 px^2^) and cholangiocyte-like cells (> 5000 px^2^) with the assumption that cells with a cytoplasm area below 5000 px^2^ are likely to be hepatocytes and those above likely to be cholangiocytes. Separation by size was further validated by anti-ASGPR1 immunostaining, a basolaterally expressed surface protein highly specific to hepatocytes (Supplementary Fig. 2). Its main function is the clearance of desialylated glycoproteins (D’Souza and Devarajan 2015). Principle component analysis was used for dimensionality reduction and performed on the hepatocyte data set and plotted using ggplot2.

### Machine learning approach to quantify apoptotic cells

Apoptotic cells were quantified using a supervised machine learning approach. Nuclei images were analyzed with CellProfiler and intensity, size, shape and texture features were extracted. These data were loaded into CellProfiler Analyst (Jones et al. 2008) together with the raw nuclei images. A random forest classifier was trained to distinguish between healthy and apoptotic cells using a training set consisting of 477 different nuclei (Supplementary Fig. 3A). To take imbalance in the dataset into account, random undersampling was performed in the training set to achieve a 67/33 ratio (323 healthy, 154 apoptotic). The training set was assembled using nuclei from the menadione exposure across three independent experiments. Apoptotic nuclei were defined based on morphological nuclear fragmentation. The model was trained until a classification accuracy between 80 and 90% was reached. Classification accuracy was determined based on user-defined assessment. In addition, images from the positive control were visually inspected to check the overall classification performance of the model and were corrected if numerous misclassifications were identified. If the classification was satisfying, the entire dataset was then scored. The resulting hit table was imported into R and the number of apoptotic cells were normalized to total cell number in the same well and further to untreated cells (Supplementary Fig. 3). Image analysis and supervised machine learning were performed on a terminal server based on Windows 10 equipped with 40 cores Intel Xeon Gold 6150 @ 2.7 Ghz and 512 GB RAM.

### Dose–response data and model fitting

Dose–response data were collected on a population level (i.e., per well averages) and combined with responses from all HepaRG cells per well. For every biological endpoint, the following raw parameters were selected per well and normalized to cell number per well:

- Lipid accumulation: Total number of lipid droplets divided by total number of cells
- Mitochondrial membrane potential: (Mean intensity–background intensity)/total number of cells
- Oxidative stress: (Mean intensity–background intensity)/total number of cells
- Nuclear morphology/cell death: number of apoptotic cells/total number of cells

These data were normalized to untreated cells to derive a fold change; unexposed cells had a fold change of 1. All data were normalized per plate to overcome possible plate effects. Non-linear regression of dose–response data was performed by fitting a log-logistic model with 3 or 4 parameters. The best fitting model was selected based on lowest Akaike Information Criterion (AIC) (Akaike 1973) and visual inspection. A positive hit, i.e., an induction of a biological endpoint by exposure, was identified when the non-linear regression exceeded the calculated threshold of ± 2 standard deviations of the response at the two lowest concentrations. Dose–response data were fitted using the drc package (Ritz et al. 2015) and plotted with ggplot2 (Hadley Wickham 2016). The R script of dose–response data and model fitting is available as supplementary material.

### Benchmark concentration modeling

To determine a point of departure of the high-content imaging concentration–response data, benchmark concentration (BMC) was modeled and calculated using the benchmark dose software (BMDS) version 3.1.2 (United States Environmental Protection Agency 2023). The benchmark dose technical guidance was used as a basis to perform the analysis (United States Environmental Protection Agency (USEPA) 2012). Normalized data including tested concentrations were entered as individual data points into the BMDS Excel application. The BMC was then defined as a benchmark response (BMR) of a change in the mean of one standard deviation from the control mean. Concentration–response data were then fitted to several models including exponential, hill, linear, polynomial and power models. Normal and log-normal distribution with constant or non-constant variance were also tested to find the best fit. BMC and its corresponding 95% confidence interval (95% CI) were then calculated. Selection criteria for the model with the best fit were the Akaike Information Criterion (AIC), goodness of fit *p*-value, scaled residuals for dose group near BMD, and for control dose group and BMDS recommendation. If there were several models which received a BMDS recommendation, BMCs from all models were averaged. BMCs were calculated for all chemicals tested (Supplementary table 3).

### In vitro to in vivo extrapolation (IVIVE)

IVIVE was performed based on the previously published method by Rotroff and Wetmore et al. (Rotroff et al. 2010; Wetmore et al. 2015) and using the high-throughput toxicokinetic (httk) R package (version 1.10.1) developed by the US-EPA. The httk package contains four toxicokinetic models which can be parameterized using high throughput-derived in vitro data on plasma protein binding and hepatic clearance. Moreover, it has a Monte Carlo sampler to simulate population variability and includes tools for reverse dosimetry together with functions for the analysis of concentration versus time simulations (Pearce et al. 2017). In our study, a three-compartment steady state pharmacokinetic (PK) model was parameterized and used for the simulations. For all simulations, the following assumptions were made: oral route of exposure, daily dosing with constant dose rate, and 100% bioavailability. Human data were used except where otherwise stated. Steady-state concentration (*C*_ss_) in the blood was calculated using formula 1 (Pearce et al. 2017)

1 $${C}_{ss} \left(\frac{\mathrm{mg}}{\mathrm{l}}\right)=ko/(\mathrm{GFR}\times {f}_{up}+\frac{\left({Q}_{\mathrm{liver}}+{Q}_{\mathrm{gut}}\right)\times {f}_{\mathrm{up}}\times {Cl}_{\mathrm{metabolism}}}{\left({Q}_{\mathrm{liver}}+{Q}_{\mathrm{gut}}\right)+\frac{{f}_{\mathrm{up}}\times {Cl}_{\mathrm{metabolism}}}{{R}_{\mathrm{blood}2\mathrm{plasma}}}})$$

where ko = constant dose rate (mg/kg BW/day), *f*_up_ = in vitro measured chemical fraction unbound in plasma, Cl_metabolism_ = metabolic clearance scaled from in vitro intrinsic hepatic clearance, *R*_blood2plasma_ = ratio of blood concentration of a chemical to the plasma concentration, GFR = glomerular filtration rate (mean 5.17 ml/min/kg^3/4), *Q*_liver_ = blood flow to the liver (mean 59.9 ml/min/kg^3/4), and Q_gut_ = blood flow to the gut (mean 47.5 ml/min/kg^3/4). Fraction unbound, metabolic clearance, and R_blood2plasma_ values were obtained from the httk package or from literature. Recognizing that formula 1 is linear in dose rate (ko), oral equivalent doses (OED) were then calculated using the *C*_ss_ predicted for a 1 mg/kg/day dose rate as in formula 2 (Rotroff et al. 2010).

2 $${\text{OED}}\left( {\frac{{\frac{{{\text{mg}}}}{{{\text{kg}}}}}}{{{\text{day}}}}} \right) = {\text{BMC}} \left( {{\text{uM}}} \right) \times \frac{{\frac{{{1}\frac{{{\text{mg}}}}{{{\text{kg}}}}}}{{{\text{day}}}}}}{{C_{{{\text{ss}}}} \left( {{\text{uM}}} \right)}}$$

The calculated OED corresponds to an oral daily dose which would lead to a plasma concentration equal to the in vitro-derived BMC. The OED is linearly related to the BMC and inversely related to *C*_ss_. This equation is only valid for first-order metabolism (Rotroff et al. 2010). A Monte Carlo analysis was used to simulate population variability in, GFR, *Q*_liver_, *Q*_gut_, and Cl_metabolism_ across 1000 healthy individuals. Extrapolations were performed for each chemical and endpoint which was positive in the imaging assay. Every predicted parameter per chemical was then combined into one boxplot per endpoint, which contained all predicted OEDs across all positive endpoints.

For the model evaluation, *C*_ss_ of selected reference chemicals were predicted using formula 1 with the same dose rate from the in vivo Css measurements. For chemicals with no in vitro intrinsic hepatic clearance and fraction unbound values available in the database included in the httk package, in silico predictions from (Sipes et al. 2017) were loaded using load_sipes2017(). In addition to that, in vitro intrinsic hepatic clearance values were calculated for a selection of chemicals (Lomitapide, Fialuridine, Metformin, beta-naphthoflavone and Menadione) using in vivo clearance values (Supplementary table 4) and formula 3:

3 $${Cl}_{\mathrm{int}}= \frac{({Cl}_{metabolism}\times {F}_{ub.corr})}{{\rho }_{\mathrm{liver}}\times {N}_{\mathrm{cells}}\times {V}_{\mathrm{liver}}\times {f}_{\mathrm{mc}2\mathrm{c}}\times {f}_{\mathrm{ml}2\mathrm{l}}\times {f}_{\mathrm{min}2\mathrm{h}}\times {f}_{\mathrm{l}2\mathrm{ul}})}$$

where Cl_int_ = in vitro intrinsic hepatic clearance in ul/min/10^6 cells, Cl_metabolism_ = in vivo clearance in L/h/kg (if available hepatic otherwise total clearance), *F*_ub.corr_ = assay correction factor which is assumed to be 1 (i.e., no corrections), *ρ*_liver_ = liver density of 1.05 g/ml, *N*_cells_ = 1.1 × 10^8 hepatocytes per gram of liver, *V*_liver_ = liver volume: 0.0245 L/kg (human), 0.0349  L/kg (rat), 0.04 L/kg (rabbit), *f*_mc2c_ = conversion factor for millions of cell to one cell, f_ml2l_ = conversion factor from ml to l, f_min2h_ = conversion factor from minute to hour, and f_l2ul_ = conversion factor from L to ul. The formula to calculate Cl_int_ was derived from the function calc_hepatic_clearance () within the httk package. Predicted *C*_ss_ values were then compared to published in vivo *C*_ss_ values. If there was no published in vivo *C*_ss_ available, *C*_ss_ was then calculated based on published in vivo pharmacokinetic parameters using the following formula (Supplementary table 3)

4 $${C}_{ss} (\frac{mg}{L})=(Dose\times F)/(Cl\times dosing interval)$$

Vice versa, predicted OED derived from BMC were compared to published exposure values of the corresponding chemicals. Simulations were performed in humans where pharmacokinetic parameters for humans were available; otherwise, estimations were calculated in rats or rabbits, as stated in Fig. 7. The R script written to perform the IVIVE analysis is available as supplementary material.

**Fig. 2:**
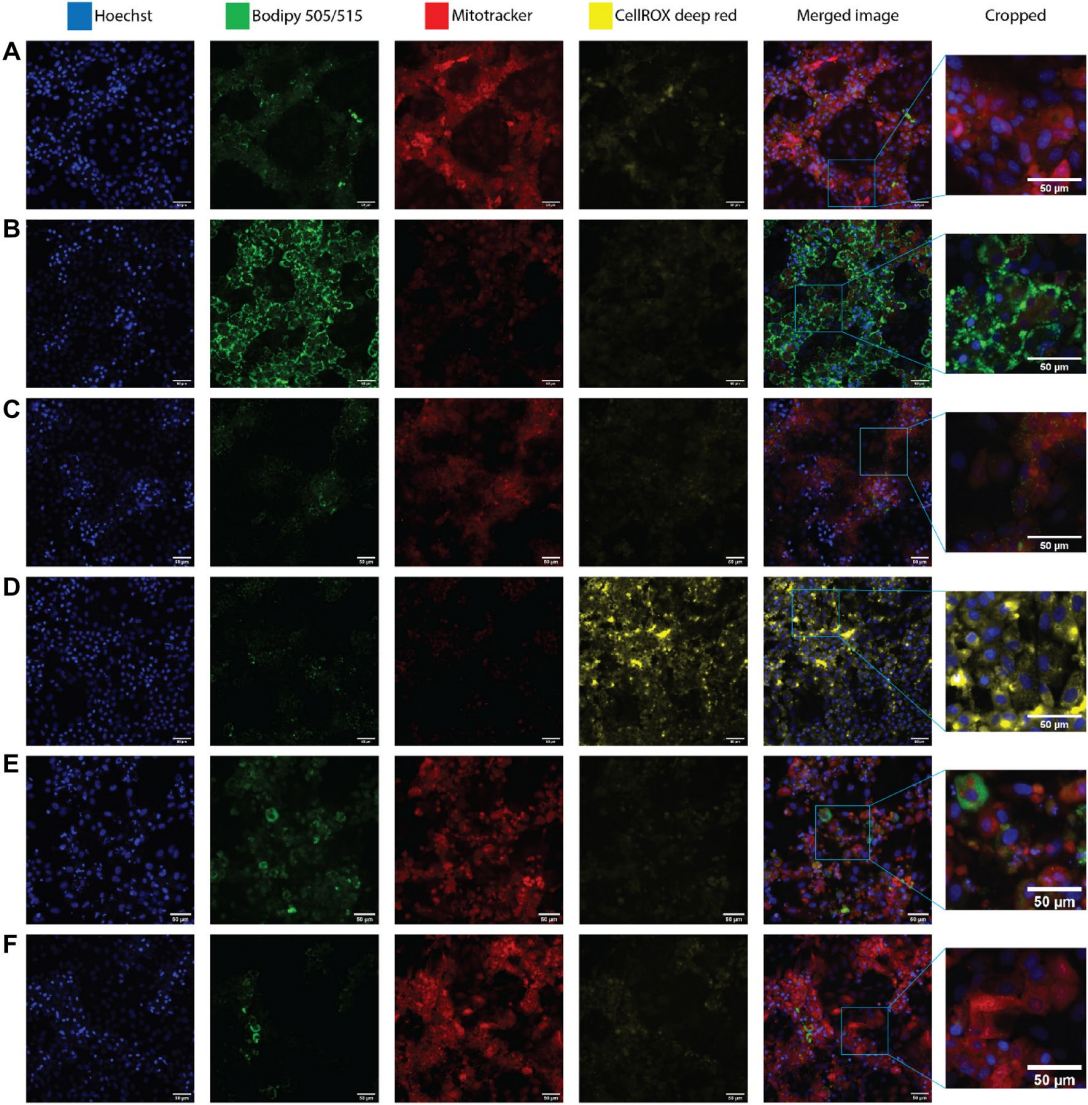
Representative fluorescent images from chemically exposed HepaRG cells. **A** DMSO control **B** Oleic acid/palmitic acid (1000 µM) **C** Rotenone (20 µM) **D** Amiodarone (50 µM) **E** Menadione (50 µM) F) Caffeine (5 mM). Uncropped images were acquired at 20x. Scalebar = 50 µm. Blue nuclei, green lipid accumulation, red mitochondrial membrane potential, yellow oxidative stress

## Results

### Multiparametric analysis of reference chemicals

To validate the assay, positive and negative control chemicals with previously established mechanisms of action were characterized by the high-content imaging approach described above (Fig. 1A). Thus, we tested a mixture of oleic acid and palmitic acid, which are often used to induce steatosis in vitro (Gómez-Lechón et al. 2007; Sharma et al. 2011; Graffmann et al. 2016; Michaut et al. 2016); rotenone, a selective complex I inhibitor (Siddiqui et al. 2013) and carbonylcyanide-p-trifluoromethoxyphenylhydrazone (FCCP), a weak acid that selectively increases proton permeability in lipid membranes (Benz and McLaughlin 1983; Sakamuru et al. 2016), were therefore used as positive controls for inducing mitochondrial dysfunction via a decrease in mitochondrial membrane potential; amiodarone and arsenite, which induce oxidative stress (Anthérieu et al. 2011; Tolosa et al. 2016; Zhao et al. 2019; Lv et al. 2020); menadione, which induces DNA damage and cell death (Loor et al. 2010); and caffeine as negative control (Persson et al. 2013; Saito et al. 2016) (Fig. 2). Concentration ranges were selected on the basis of preliminary cell viability screen, and each chemical was used at concentrations ranging up to the corresponding EC_50_ value (Supplementary Fig. 4).

**Fig. 3:**
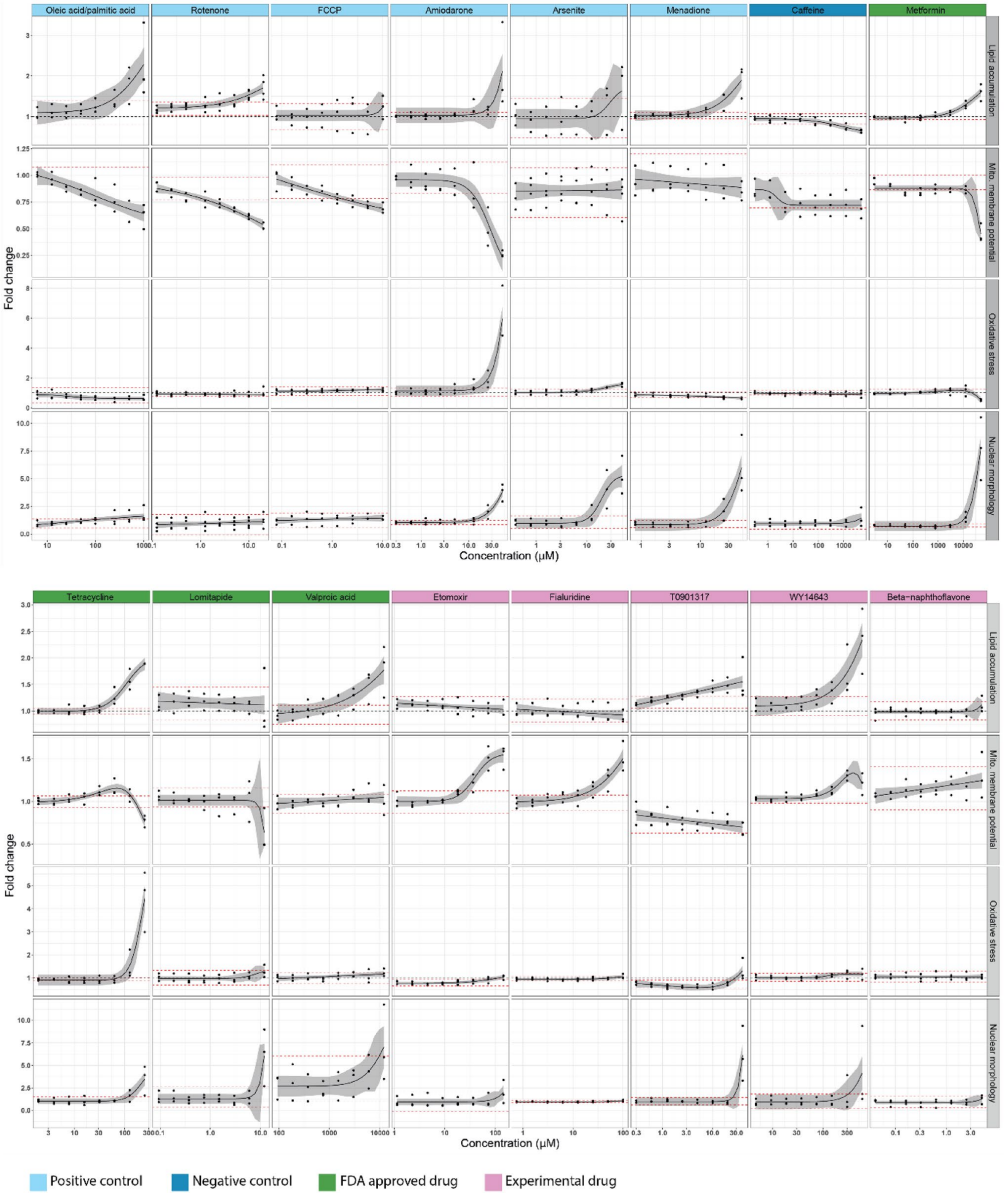
Cellular responses for four key steatosis-relevant endpoints. Gray area around non-linear regression represents 95% CI. Black dotted line represents a fold change of 1 (DMSO control). Red dotted lines represent threshold (± 2 standard deviations of the response at the two lowest concentrations). All data were first normalized to cell number and then to untreated cells. At least 3 independent experiments with 6 technical replicates per concentration per experiment were conducted for all chemicals

Response profiles of the positive and negative control compounds were consistent with expected outcomes. Lipid accumulation increased in a dose-dependent manner upon exposure to oleic acid/palmitic acid, rotenone, amiodarone and menadione, with oleic acid/palmitic acid (1000 µM) being the most efficacious (2.3-fold increase). Caffeine induced a decrease of lipid accumulation that exceeded the threshold, but only at the highest concentration (5000 µM). In the case of mitochondrial membrane potential, oleic acid/palmitic acid (62.5 µM), rotenone (2.5 µM), fccp (2.5 µM) and amiodarone (25 µM) all induced a dose-dependent decrease exceeding the threshold at the concentrations indicated. In the case of oxidative stress, amiodarone (25 µM) and arsenite (25 µM) exposure led to a dose-dependent increase and exceeded the threshold at the concentrations indicated. The number of apoptotic cells (nuclear morphology) increased in oleic acid/palmitic acid-(250 µM), amiodarone- (25 µM), arsenite- (25 µM) and menadione- (25 µM) treated HepaRG cells in a dose-dependent fashion, exceeding the threshold at the concentrations indicated (Fig. 3). These observations are consistent with previous response patterns and sensitivities (Anthérieu et al. 2011; Tolosa et al. 2016; Angrish et al. 2017; Allard et al. 2020) (Gómez-Lechón et al. 2007; Anthérieu et al. 2011; Sharma et al. 2011; Siddiqui et al. 2013; Graffmann et al. 2016; Michaut et al. 2016; Tolosa et al. 2016), suggesting the accuracy of the in vitro assay and data analysis pipelines.

Following the effective systematic characterization of positive and negative control chemicals for each endpoint, we tested four Food and Drug Administration (FDA)-approved drugs with known mechanisms of action. They included metformin, an anti-diabetic drug that inhibits mitochondrial complex I (Pernicova and Korbonits 2014), tetracycline, an antibiotic drug that inhibits β-oxidation, microsomal triglyceride transfer protein (MTP) and upregulates PPARγ and SREBP1-c (Anthérieu et al. 2011; Schumacher and Guo 2015), lomitapide, an anti-hypercholesterolemic drug that inhibits MTP (Lin et al. 2014), and valproic acid, an anti-convulsant drug that inhibits β-oxidation and upregulates CD36 and DGAT2 (Schumacher and Guo 2015; Bai et al. 2017). Lipids accumulated in a dose-dependent manner above the threshold for metformin, tetracycline and valproic acid, but not lomitapide. Mitochondrial membrane potential decreased when cells were exposed to metformin (50,000 µM) or lomitapide (12 µM), but only at the highest concentrations. An unexpected hormetic effect was observed in cells exposed to tetracycline (between 31.25 and 125 µM); a hyperpolarization, which to our knowledge has not been reported previously, was observed, such that only at the highest concentration (250 µM) did the mitochondrial membrane potential decrease. Oxidative stress dose-dependently increased after tetracycline (125 and 250 µM) exposure and exceeded the threshold at the concentrations indicated, but no changes were observed for lomitapide or valbroic acid. A unique decrease of oxidative stress below the threshold was observed after metformin exposure (50,000 µM). Finally, the number of apoptotic cells increased above the threshold in metformin, tetracycline and lomitapide treated cells (Fig. 3). These data thus confirmed the anticipated activation profiles of the cellular markers for these reference compounds.

As a next step, we were interested to test etomoxir and fialuridine, both chemicals that had their clinical development terminated due to severe hepatotoxicity in clinical trials, but with limited knowledge of the underlying mechanisms (Manning and Swartz 1995; Holubarsch et al. 2007). Etomoxir was developed as an anti-diabetic drug and is an irreversible carnitine palmitoyltransferase 1 (CPT1) inhibitor (Merrill et al. 2002). Fialuridine is a nucleoside analog that was developed to treat chronic hepatitis and inhibits mitochondrial DNA polymerase γ (McKenzie et al. 1995; Lewis et al. 2003). However, neither induced lipid accumulation, had any consistent impact on oxidative stress, nor induced apoptosis (Fig. 3). Nonetheless, fialuridine (25 µM) and etomoxir (36 µM) induced an increase in MMP and exceeded the threshold the concentrations indicated (Fig. 3). The profiles for these known steatotic compound, i.e., lack of lipid accumulation with MMP increase, illustrate a concept behind using a hallmark-driven assay and potential difficulties in modeling lipid accumulation after short in vitro exposures. Thus, chemicals that induce related measures of hepatotoxicity, which may be easier to detect, may be flagged for more in-depth evaluation with a focus on lipid accumulation potential, such as longer duration exposures.

We next were interested in testing chemicals known to bind to nuclear receptors, as nuclear receptor activation is a molecular initiating event of steatosis. Thus, we characterized three experimental drugs: T0901317, a synthetic liver X receptor agonist that increases the expression of SREBP-1c and CHREBP (Mitro et al. 2007; Cha and Repa 2007); WY14643, a dual PPARα/PPARγ agonist that increases oxidative stress in mouse liver and affects fatty acid metabolism (Woods et al. 2007; Jennings et al. 2014); and β-naphthoflavone (BNF), an aryl hydrocarbon receptor agonist that upregulates CD36 (Lee et al. 2010; Jennings et al. 2014). Lipid accumulation dose-dependently increased and exceeded the threshold for cells exposed to T0901317 and WY14643, but not BNF. In addition to inducing lipid accumulation, T0901317 caused a decrease in MMP below the threshold, and oxidative stress and apoptosis exceeding the threshold at the highest T0901317 concentration (40 µM). For WY14643, which also induced lipid accumulation, we observed MMP hyperpolarization and it induced apoptosis Yet, none of the hallmarks were induced by BNF (Fig. 3). Thus, 2 out of 3 nuclear receptor agonists induced several steatosis and hepatotoxicity related cellular events.

### Analysis of food-related chemicals and pesticides

**Fig. 4:**
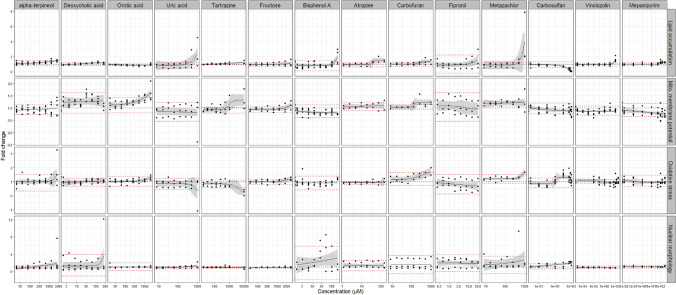
Cellular responses for four key steatosis-relevant endpoints of food-related chemicals. Gray area around non-linear regression represents 95% CI. Black dotted line represents a fold change of 1 (DMSO control). Red dotted lines represent threshold (± 2 standard deviations of the response at the two lowest concentrations). All data were first normalized to cell number and then to untreated cells. 2-3 independent experiments with 6 technical replicates per concentration per experiment were conducted for all chemicals

With nuclear receptor activation as a potential initial predictor of steatotic potential, as further supported by the T0901317 and WY14643 results, we wanted to characterize more broadly environmentally relevant compounds with structural alerts for nuclear receptor binding. Thus, about 6,000 Smiles (Simplified Molecular Input Line Entry System, a chemical language system in which chemical structures can be written using ASCII characters (Weininger 1988)) were extracted from the regulated food-related use/occurrence chemical database from the Swiss Federal Food Safety and Veterinary Office and about 4,000 from the Toxcast database (Richard et al. 2016). We assessed potential for nuclear receptor binding on the basis of molecular fragments and other relevant chemical features, by a previously reported approach (Mellor et al. 2016), and identified about 80 chemicals that might bind one or more receptors. Of these 80 compounds, 14 had prior evidence of hepatotoxicity and published toxicokinetic data (Supplementary Table 6), and were selected for further testing in the assay established in this study. Of these 14 food-related chemicals and pesticides, six of them (uric acid, tartrazine, bisphenol A, atrazine, metazachlor, and vinclozolin) induced significant lipid accumulation. Orotic acid, fructose and carbofuran did not stimulate lipid accumulation, but did stimulate a dose-dependent increase in MMP exceeding the threshold. No response was observed in oxidative stress or the number of apoptotic cells after 24 h exposure (Fig. 4). Thus, 9 out of 14 food-related chemicals and pesticides induced steatosis and/or an increase in MMP, whereas none induced oxidative stress or an increase in the number of apoptotic cells.

### Chemical exposures induce distinct cellular endophenotypes within cell populations

**Fig. 5:**
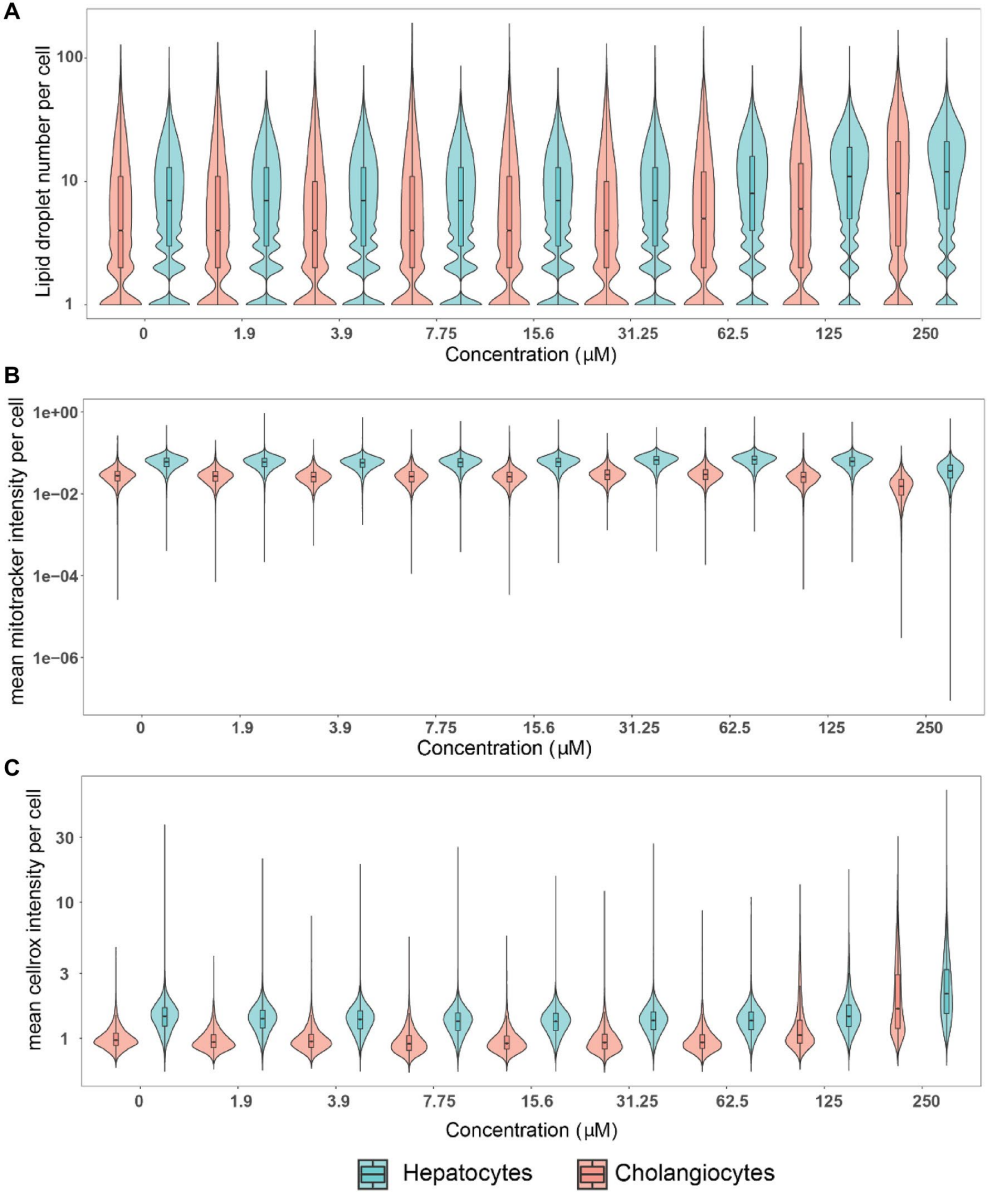
Quantification of selected endpoints in individual hepatocytes versus cholangiocyte-like cells after tetracycline exposure. The same raw data used in the population-level analysis were also used for this single cell analysis. 30,000 to 150,000 cells per concentration and cell type were analyzed. Cells from 3 independent experiment were included in the analysis. Notched boxplots were overlaid in the violin plots to display the confidence interval around the median

To evaluate the potential heterogeneity of single cell responses and interrogate potential endophenotypes within and/or between cell populations in the HepaRG cells, we further evaluated alterations of lipid accumulation, mitochondrial membrane potential and oxidative stress following tetracycline exposure. Tetracycline was selected because it induced responses above the threshold in all four endpoints. First of all, we observed that in general, there were more lipid droplets in hepatocyte-like cells compared to cholangiocyte-like cells even in unexposed cells (Fig. 5), and, moreover, at 125 µM and 250 µM, the median number of lipid droplets per hepatocyte-like cell did not drastically increase (~ 8 to ~ 12 lipid droplets per cell), but the proportion of hepatocyte-like cells with more lipid droplets increased. The opposite response was observed for cholangiocyte-like cells: the median number of lipid droplets per cholangiocyte-like cells increased, but the proportion of cholangiocyte-like cells with more lipid droplets remained unchanged (125 µM or 250 µM tetracycline, Fig. 5). Similar to lipid droplets, the mitochondrial membrane potential also increased more in hepatocyte- compared to cholangiocyte-like cells for all tetracycline concentrations tested. Notably, at the highest concentration of tetracycline (250 µM), a decrease of mitochondrial membrane potential was observed in both cell types. Finally, for oxidative stress, similar patterns of changes were observed in hepatocyte-like cells which had increased levels of oxidative stress compared to cholangiocyte-like cells throughout all tested concentrations. At the highest concentration at which tetracycline was tested (250 µM), oxidative stress levels were elevated in both cell types. By evaluating increased oxidative stress on a single cell basis, it became apparent that the increased average values for oxidative stress on a cell population level arose from a small number of cells with extremely high levels of oxidative stress, rather than constitutive moderate increase across the whole cell population (Fig. 5). In summary, hepatocyte-like cells appear to be more sensitive than cholangiocyte-like cells and that population-level responses appear to be driven by the evolution of small populations with high responses rather than an equal distribution of responses amongst cells.

**Fig. 6:**
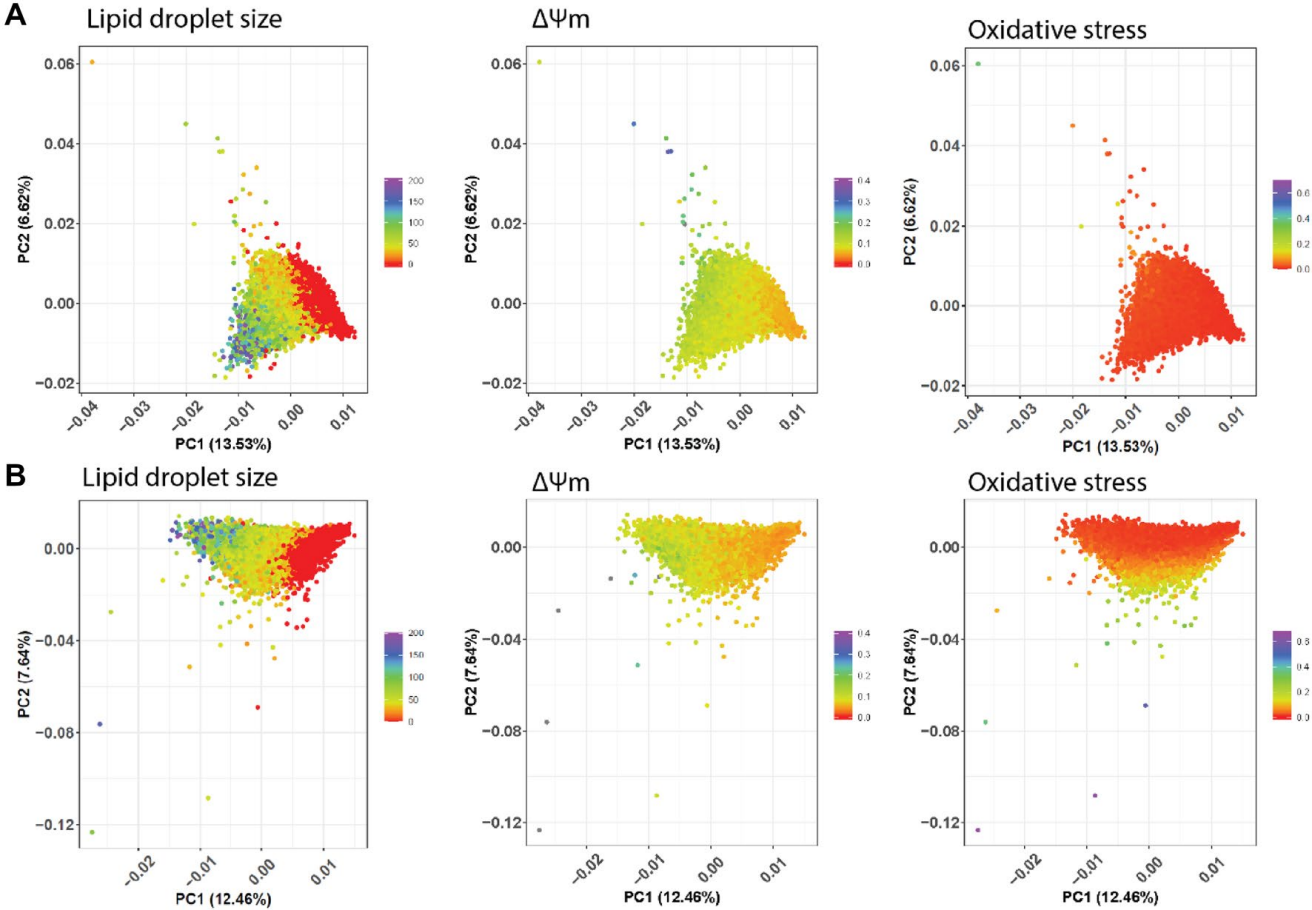
Heterogenous response pattern after tetracycline exposure in hepatocytes. **A** untreated **B** tetracycline (250 µM). A single point in the PCA plot represents a hepatocyte and its color code represents lipid droplet size (px^2), mitochondrial membrane potential (ΔΨm) (AU) or oxidative stress (AU). 80,000 to 125,000 cells from 3 independent experiments were analyzed per concentration

Given the heterogeneity of responses in hepatocyte- and cholangiocyte-like cells, we investigated whether hepatocyte-like cells with a strong response in one endpoint (e.g., large lipid droplet size) responded similarly in other endpoints (mitochondrial membrane potential and oxidative stress) at the same concentration. For this purpose, over 800 features per cell were extracted by high-content imaging and resulting PCA plots were color labeled with lipid droplet size, mitochondrial membrane potential, and oxidative stress level to visualize their distribution (Fig. 6). Heterogeneity in lipid droplet size and mitochondrial membrane potential was observed in both chemically and mock-exposed hepatocyte-like cells, whereas it was only observed for oxidative stress in chemically exposed cells. Oxidative stress levels in non-exposed hepatocytes were similar across all measured hepatocytes (Fig. 6A). While mitochondrial membrane potential did not decrease in hepatocytes with larger lipid droplets it did decrease in hepatocytes with smaller lipid droplets (Fig. 6B). Similarly, oxidative stress levels in chemically exposed hepatocytes with larger lipid droplets were lower than in hepatocytes with medium and small sized lipid droplets. Interestingly, a subgroup of hepatocytes with medium and small sized lipid droplets also had lower oxidative stress levels compared to other hepatocytes with the same lipid droplet size. Thus, tetracycline exposure led to heterogenous response patterns in hepatocytes involving a subpopulation that was protected from mitochondrial dysfunction and oxidative stress.

#### IVIVE

**Fig. 7:**
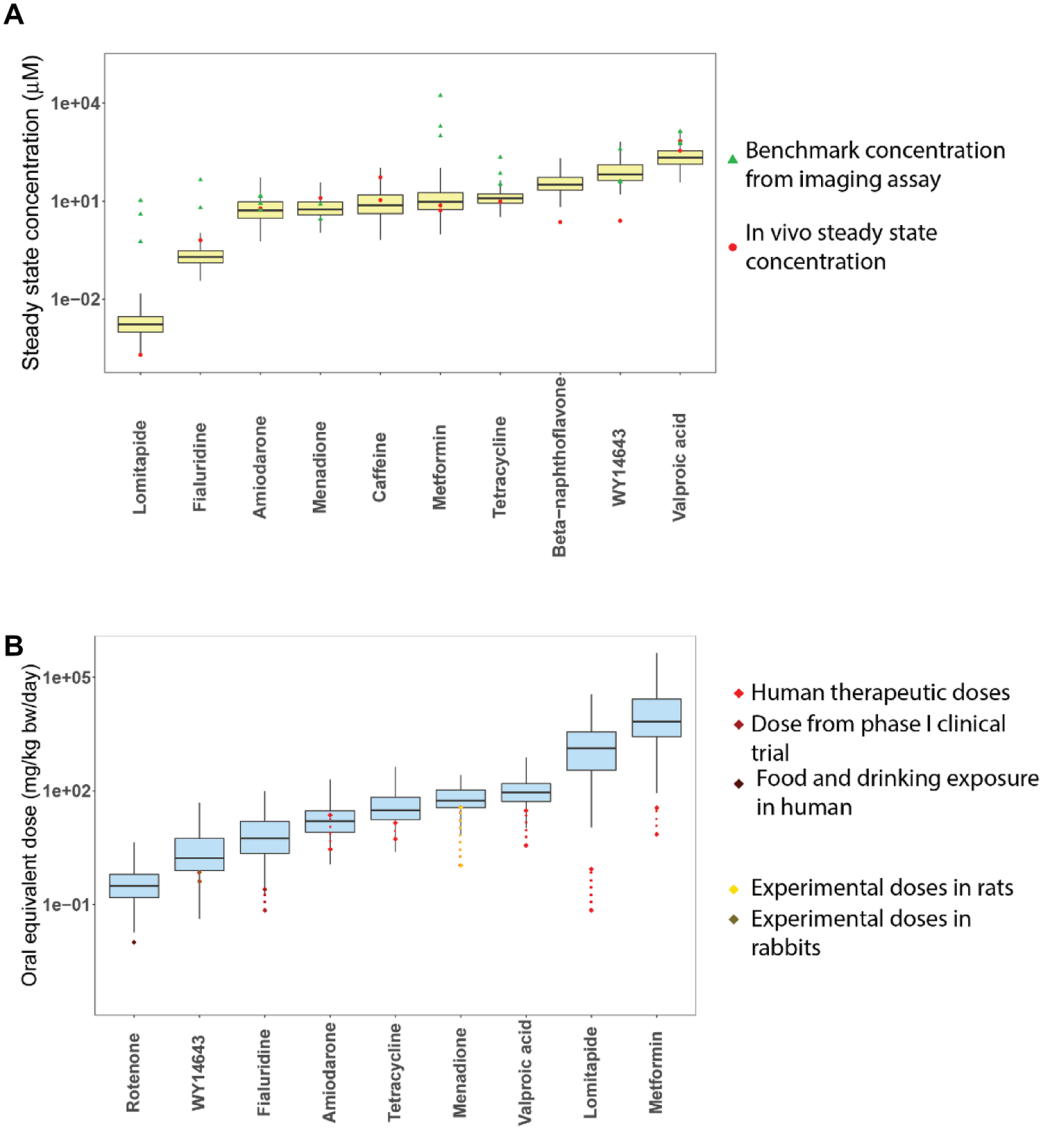
Pharmacokinetic modeling of selected reference chemicals. **A** Forward dosimetry of chemicals with published Css values. Yellow boxplot represents predicted Css using IVIVE. **B** Reverse dosimetry of chemicals with published human exposure values from different sources. Blue boxplot represents predicted oral equivalent doses using httk with a monte carlo population sampler. OED predictions were performed for every endpoint from the HCI assay which was above the calculated threshold

We used pharmacokinetic modeling and reverse dosimetry to evaluate the relevance of the in vitro concentrations that stimulated one or more of the endpoints defined as exceeding a calculated threshold of ± two times the standard deviations of the response to the two lowest concentrations. Predicted *C*_ss_ values were compared with in vivo *C*_ss_ values (Fig. 7A), which were derived from pharmacokinetic studies in human, rat or rabbit (Supplementary table 3). For the ten chemicals, the coefficient of variation (*R*^2^) on a logarithmic scale was 0.78 and the root mean squared error was 0.69 (a factor of 4.9x). The chemicals with the greatest discrepancy between actual and predicted *C*_ss_ were beta-naphthoflavone and WY-14643, where the predicted *C*_ss_ were about 13 to 26 times higher than the actual *C*_ss_ values.

Benchmark concentrations (BMC) were then calculated for every positive response indication in the imaging assay to determine a point of departure (Supplementary table 3 and Table 1). A direct comparison between the in vitro-derived BMC and in vivo *C*_ss_ was then made to identify a potential in vivo hazard. For menadione, the BMC was about 1.5–4 times lower than the median predicted *C*_ss_. For amiodarone and valproic acid, it was about 1.5–4 times higher and for artrazine about double the predicted *C*_ss._. For orotic acid, uric acid, carbofuran, and carbosulfan, the calculated BMC was about 10–550 times higher than the predicted Css. For lomitapide, fialuridine, WY14643, metformin, and tetracycline, the BMC was about 10–10,000 times higher than the median PBPK-predicted *C*_ss_. This direct comparison of in vitro-derived BMC with in vivo *C*_ss_ was consistent with the indication of menadione, but only this compound, which is a known hepatotoxicant, was identified as a hazard.

**Table 1:** IVIVE of selected food-related chemicals which showed a response above the calculated threshold in the imaging assay

Chemical	LA^1^	MMP^1^	OS^1^	NM^1^	Median Css^2^ (µM)	In vivo blood plasma conc. (µM)	OED^3^ (mg/kg BW/day)	NOAEL/LOAEL from literature (mg/kg BW/day)	Exposure estimate from literature	Maximum residue levels (mg/kg)	Reference
Orotic acid	–	423	–	–	7.04	0.15–0.3	61.2 (95% CI 21.1–134)	50/100	2–100 mg/kg BW/day	–	(Aguilar et al. 2009; D’Apolito et al. 2012)
Uric acid	591	–	–	–	6.84	217–402	87.3 (95% CI 67.3–150.6)	–	–	–	(Li et al. 2009)
Atrazine	19	–	–	–	40	–	0.5 (95% CI 0.2–1.7)	5/50 (increase relative liver weight)	–	0.05–0.1	(Agency for toxic substances and disease registry ATSDR 2003)
Carbofuran	–	4975	–	–	9	–	565 (95% CI 91.7–infinity	0.03–0.1/0.1–0.3	–	0.01–0.5	(Food and Authority 2009a)
Carbosulfan	77	–	–	–	8.5	–	10.4 (95% CI 7–19.3)	0.5–1.2/5–62	–	0.005	(Food and Authority 2009b)

*LA* lipid accumulation, *MMP* mitochondrial membrane potential, *OS* oxidative stress, *NM* nuclear morphology, ^1^Benchmark concentration (µM) per imaging endpoint calculated with BMDS software, ^2^Derived from in vitro to in vivo extrapolation, ^3^In vitro to in vivo extrapolation derived median

As the *C*_ss_ metrics were not available for all the chemicals, we used a three-compartment steady state PK model to derive oral equivalent doses (OED) from respective BMC values. OEDs were predicted for chemicals with available exposure data such as human therapeutic doses, doses from clinical trials, food and drinking amounts, or experimental doses in animal models. OED was then predicted for every endpoint for which a significant response was observed in the imaging assay (Fig. 7 and Tables 1 and 2). Predicted OED values overlapped with realistic doses for the drugs fialuridine, tetracycline, amiodarone, valproic acid and menadione, whereas for Rotenone, WY14643, Lomitapide and Metformin, the OED values were 10–10,000 times higher than the doses. The OED values for orotic acid and carbosulfan overlapped with their reported no observable adverse effect level (NOAEL), lowest observed adverse effect level (LOAEL) concentrations, or the exposure estimate.

**Table 2:** Comparison of predicted oral equivalent doses (OED) with published exposure values

Chemical	OED^a^ (mg/kg/d)	In vivo exposure from literature^b^ (mg/kg/d)
Rotenone	0.3	0.01^c^
Fialuridine	5.6	0.07–0.25^d^
Amiodarone	15.8	2.9–22.9^e^
Tetracycline	30.2	5.4–14.3^f^
WY14643	1.7	0.4–0.7^ g^
Valproic acid	92.7	3.6–30^ h^
Menadione	55.4	1.1–36.6^i^
Lomitapide	1333	0.07–0.86^j^
Metformin	6778	7.1–35.7^ k^

^a^In vitro to in vivo extrapolation derived median

^b^Exposure values include human therapeutic ranges or doses given to rat and rabbits

^c^(United States Environmental Protection Agency (USEPA) 2007)

^d^(Kleiner et al. 1997)

^e^(Siddoway 2003)

^f^(Bienenfeld et al. 2017)

^g^(Pollinger and Merk 2017)

^h^(Koch-Weser and Browne 1980)

^i^(Hu et al. 1996)

^j^(Panno et al. 2014)

^k^(Garber et al. 1997)

## Discussion

**Fig. 8:**
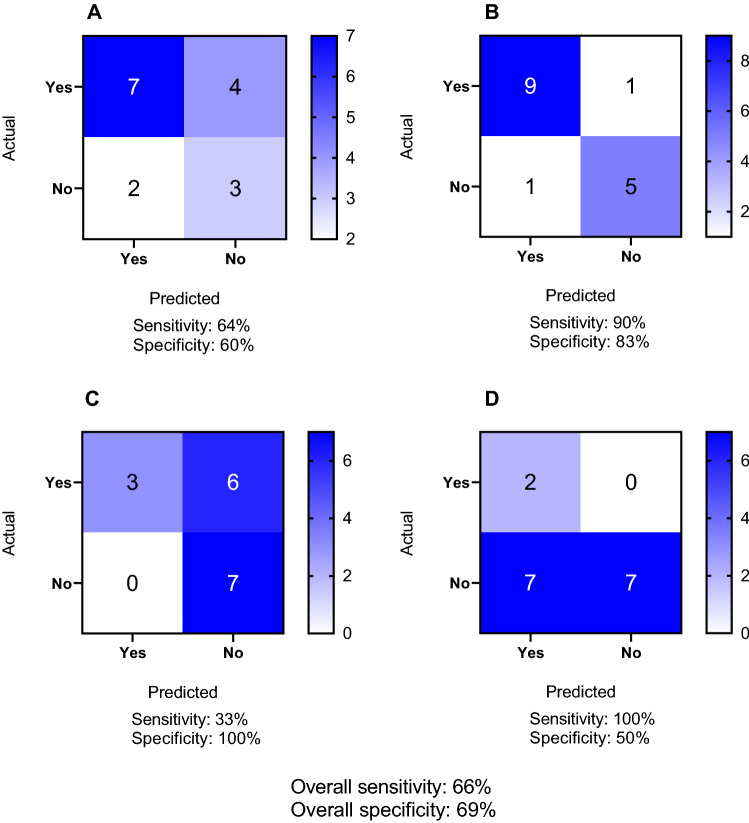
Quantification of the predictivity of the in vitro assay for **A** lipid accumulation **B** mitochondrial membrane potential **C** oxidative stress and **D** nuclear morphology for 16 reference chemicals using confusion matrices. Sensitivity was calculated as “number of true positives/(number of true positives + number of false negatives)” and specificity was calculated as “number of true negatives/(number of true negatives + number of false positives)”. Overall sensitivity and specificity were calculated across all 4 endpoints. Of the 16 reference compounds tested, the outcomes were categorized as negative or positive, excluding amongst positive outcomes instances where no dose–response relationship was observed

In this study we combined a high-content imaging assay for chemically exposed metabolically competent human liver cells with PBPK modeling to derive relevant human exposure predictions for the potential of chemicals to induce hepatic steatosis or other hepatotoxic processes. Validation studies were carried out using 16 reference chemicals with an overall sensitivity of 66% and specificity of 69% (Fig. 8). Notably, 6/9 predicted OED values for selected reference chemicals overlapped with estimated exposure values. Orotic acid disrupted MMP, but not other markers, at a concentration corresponding to an OED that overlaps with estimated human exposures. These outcomes are notable for the high accuracy of predicted OED with human exposure values for known chemicals, and the application of the method to screen food-related chemicals.

In this study we correctly flagged 7/11 chemicals known to induce lipid accumulation, and also identified two chemicals, metformin and WY-14693, as having the potential to induce lipid accumulation, that were unexpected (Fig. 8). A potential rationale for this observation, in the case of metformin, is that it induced lipid accumulation only at extremely high, physiologically irrelevant concentrations (25,000 and 50,000 µM), whereas data suggesting that metformin reduces lipid accumulation was observed for concentrations ranging from 100 to 2 mM (Zare et al. 2019; Kim et al. 2020). Furthermore, four chemicals, lomitapide, etomoxir, fialuridine and β-naphthoflavone did not give rise to lipid accumulation as we would have expected based on their established mechanism of action and previous clinical observations (Supplementary table 2) (McKenzie et al. 1995; Cuchel et al. 2013). A possible explanation for these apparent false negative results is that the 24-h duration of exposure may be too short for these chemicals to induce lipid accumulation. Indeed, lipid accumulation in patients was observed only after 13 weeks for fialuridine and 26 weeks for lomitapide (McKenzie et al. 1995; Cuchel et al. 2013). This limitation suggests that future research using the assay reported here, but beyond the scope of the present work, should aim to evaluate how longer duration in vitro exposures, i.e. on the order of one to two weeks may resolve false negative results. Encouragingly, in previous studies, hepatotoxicity was effectively characterized in HepaRG cells after up to 14 days of chemical exposure (Anthérieu et al. 2011; Dietrich et al. 2020; Donato et al. 2022).

In addition to lipid accumulation data, we could assess changes in mitochondrial membrane potential, oxidative stress and nuclear morphology with varying degrees of accuracy (Fig. 8). Thus, in the case of the 10 chemicals expected to disrupt MMP, 9 were indeed positive in our assay, whereas one chemical, valproic acid, did not affect MMP as we would have expected based on its mechanism of action (Supplementary table 2) (Fig. 8). This is potentially expected since valproic acid has been characterized to induce mitochondrial dysfunction at very high concentrations, i.e. above 15 mM, and after prolonged exposure up to 72 h (Caiment et al. 2020) whereas we tested concentrations ranging from 94 to 6,000 µM and exposed the cells for 24 h. Interestingly, etomoxir and fialuridine also increased MMP, which to our knowledge has not been reported previously. However, the biological relevance of this observation is still unclear and needs further investigation.

In the case of oxidative stress, sensitivity was poor; of the 9 chemicals expected to induce oxidative stress, only amiodarone, arsenite and tetracycline actually did, while six were unexpected negative outcomes, including the well-known ROS-inducer menadione (Fig. 8). This apparent low sensitivity could also be due to potential duration differences and a capacity for adaptive responses since menadione induced oxidative stress in embryo chick cardiomyocytes after exposure durations as short as 25 min (Loor et al. 2010). Thus, in addition to longer than 24 h durations mentioned previously, also shorter exposure durations than 24 h could be important to provide a more dynamic picture of oxidative stress responses in hepatocytes. While the simultaneous detection of various markers is highly appealing, there is also a limitation that the dynamics of various processes arise following different durations. Finally, nuclear morphology appeared to be an over-sensitive read-out in this assay. Whereas only two chemicals were expected to induce such changes (arsenite and menadione), altered nuclear morphology was actually observed for a total of 8 chemicals. Nuclear morphology response mostly was above the calculated threshold at the highest tested concentrations, consistent with cell viability data (Supplementary Fig. 4).

Following the characterization of the various reference chemicals, we applied the assay to test 14 food-related chemicals, 6 of which induced steatosis. Since the test chemicals were selected from a large number of chemicals with a focus on predictions of receptor-binding potential, it is not surprising that so many induced steatosis, however, at high concentrations relative to real exposures. Interestingly, orotic acid did not induce steatosis but disrupted mitochondrial membrane potential at extrapolated doses overlapping with actual exposure values (Table 2). Indeed, orotic acid is known to induce fatty liver in rats, whereas other animal species including mice and monkeys did not develop fatty liver upon orotic acid exposure (Durschlag and Robinson 1980; Löffler et al. 2015). These findings are consistent with a concern that orotic acid might be a potential risk factor for hepatotoxicity and steatosis in humans, however further investigations are needed. For the other two endpoints, namely oxidative stress and apoptosis, none of the tested compounds appeared to induce responses. This may be related to the selection criteria, which was not directly related to associated molecular initiating events, or may be related to the requirement for longer exposure duration to stimulate these further cellular effects.

In vitro response data typically describe cell population average responses, missing potentially relevant information about how sub-populations might react to chemical exposure (Singh et al. 2014). In our study, we performed single cell analysis and investigated how hepatocytes and cholangiocyte-like cells responded on a single cell level to tetracycline exposure. For tetracycline in particular, we could readily observe from preliminary measurements that responses of individual cells were heterogeneous, so we quantified the distribution. To our knowledge, this is the first time that chemically exposed HepaRG cells were analyzed on a single cell level, discriminating cellular responses in hepatocyte and cholangiocyte-like cells. Interestingly, hepatocytes always responded more than cholangiocyte-like cells, possibly because hepatocytes may take up chemicals more efficiently than cholangiocyte-like cells do. Furthermore, hepatocytes with larger lipid droplets were rather protected from a decrease in mitochondrial membrane potential compared to hepatocytes with small- and medium-sized lipid droplets. Likewise, hepatocytes with larger lipid droplets tended to have lower oxidative stress levels than hepatocytes with smaller ones suggesting a protective effects of larger sized lipid droplets (Jarc and Petan 2019). Further mechanistic evidence showed that lipid droplet formation may protect cells against fatty acid-induced lipid toxicity. For example, Listenberger et al. showed that excess palmitate, which could not be stored in lipid droplets, induced apoptosis (CHO cells) whereas cells were protected from apoptosis when palmitate was integrated into triglycerides and stored in lipid droplets (Listenberger et al. 2003). Furthermore palmitic acid decreased mitochondrial membrane potential in HepG2 and THP-1 cells (Li et al. 2018; Alnahdi et al. 2019). Tetracycline is known to decrease β-oxidation and free fatty acids efflux via MTP inhibition. This could lead to palmitic acid accumulating in the cytoplasm and thus further increase ROS and decrease mitochondrial membrane potential. Hepatocytes, which appear to have the capacity to shuttle excess palmitic acids into lipid droplets, may be protected from such stress events. This kind of analysis can be extended and used as a basis to formulate new hypothesis about mechanisms of toxicity.

PBPK models can be used to perform in vitro to in vivo extrapolation and predict possible human exposure scenarios that connect observed in vitro effects with human exposure levels via predicted OED values (Blaauboer 2010). In our study, predicted Css values overlapped with Css values observed in vivo for all chemicals except BNF and WY14643 (Fig. 7A). Importantly, for compounds with available human exposure data, estimated OEDs were within the range of published exposure values. Nevertheless, nominal in vitro concentrations for selected chemicals were higher compared to in vivo *C*ss (e.g., metformin or valproic acid), leading to a high estimate of the OED. The following assumptions were made during IVIVE performed in this study: (1) Constant dose rate of 1 mg/kg/d with complete absorption for every chemical and excretion was limited to the renal route; (2) Plasma protein binding was not considered when in vitro assay concentrations were used for deriving BMC; (3) Metabolism of the chemicals was not accounted for. Aside from the quantitative aspects, there are also uncertainties associated with IVIVE regarding translation of whether cellular effects observed in vitro also occur in vivo, or whether adaptive or further detrimental responses at the organ level are relevant. Quantitative relationships between higher level key events in the AOP are needed to further link the cellular phenotypes with human disease. This would need to be further validated in more complex and physiologically relevant models and compared to relevant human exposures, including in susceptible populations, to inform risk assessment.

While the use of HepaRG cells offers benefits of metabolic competency and reproducibility, there are limitations. For example, they were derived from a single donor, making it impossible to study population variability. In addition, despite being metabolically active, they do not express high levels of CYP2D6, an enzyme responsible for about 25% of drug metabolism, due to being derived from a poor CYP2D6 metabolizer patient (Guillouzo et al. 2007). Finally, they are differentiated using DMSO, a histone deacetylase inhibitor that may interfere with drug metabolism (Wang et al. 2019). In further studies, therefore, the approach may be adapted for more complex in vitro models such as primary human hepatocytes from donor pools or from multiple single donors, sandwich cultures, liver spheroids from primary human cells (including hepatocytes, Kupffer cells and hepatic stellate cells), liver-on-a-chip or body-on-a-chip models which combine liver spheroids with additional tissues such as adipose tissue or the gut with/without bacterial co-cultivation to potentially improve the prediction accuracy and biological relevance of the hepatic in vitro models to progressive NAFLD.

The results presented here comprise a new method combining high-content imaging of chemically exposed human liver cells and IVIVE to prioritize chemicals for further evaluation for their potential to induce steatosis and hepatotoxicity. Further refinement of the specificity and sensitivity of the in vitro model and methodology may include measuring additional key events related to the liver steatosis AOP such as ER stress, fatty acid influx/efflux or triglyceride synthesis, adapting the approach for high-throughput screening, testing more types of chemicals (including mixtures of chemicals), and evaluating the temporal dynamics of shorter/longer exposure scenarios. Of 14 food-relevant exposures and pesticides tested with this method, a number could induce lipid accumulation, but at levels outside the range of realistic exposures, and none induced oxidative stress or cell death, suggesting the need to screen larger numbers of compounds, at varying durations. Nonetheless, of these compounds, orotic acid was flagged for its capacity to disrupt mitochondrial membrane potential. Thus, aside from expanding the scope of screening, these apical endpoint investigations could be combined with molecular mechanistic data derived from more in-depth transcriptomic and proteomic analyses to identify additional predictive key events and refine mechanistic understanding. Future testing of existing chemicals of concern and emerging chemicals would support a first step in a tiered approach for next generation chemical risk assessment. Furthermore, this approach also could be used in a drug discovery pipeline to screen out molecules that could induce steatosis and/or unwanted changes in the additional hepatotoxicity markers, as well as to gain mechanistic insights for candidate molecules with preclinical in vivo liver steatosis findings.

## Supplementary Information

Below is the link to the electronic supplementary material.

Supplementary file1 (ZIP 3329 KB)

## Data Availability

Data are available from the corresponding author upon request.
