# Supplementary material for: In vitro to in vivo extrapolation and high-content imaging for simultaneous characterization of chemically induced liver steatosis and markers of hepatotoxicity
Source: Arch Toxicol. 2023 Apr 12;97(6):1701–21. doi: 10.1007/s00204-023-03490-8 (PMC10182956; doi:10.1007/s00204-023-03490-8)
Supplement: Supplementary file 1 — Supplementary file1 (ZIP 3329 KB) [file 204_2023_3490_MOESM1_ESM.zip › AoT_Supplementary figures_sub_final.docx]

**Supplementary information**

for

**In vitro to In vivo extrapolation and high content imaging for simultaneous characterization of chemically induced liver steatosis and markers of hepatotoxicity**

Fabrice A. Müller^1^, Marianna Stamou^1^, Felix H. Englert^1^, Ole Frenzel^1^, Sabine Diedrich^1^, Laura Suter-Dick^3,4^, John F. Wambaugh^2^ and Shana J. Sturla^1^

^1^Department of Health Sciences and Technology, ETH Zurich, 8092 Zurich, Switzerland

^2^Center for Computational Toxicology and Exposure, Office of Research and Development, United States Environmental Protection Agency, Research Triangle Park, North Carolina 27711, United States

^3^Schoof of Life Sciences, University of Applied Sciences and Arts Northwestern Switzerland, 4132, Muttenz, Switzerland

^4^Swiss Centre for Applied Human Toxicology (SCAHT), 4001, Basel, Switzerland

Corresponding author: Prof. Dr. Shana J. Sturla, ETH Zurich, Department of Health Sciences and Technology, Schmelzbergstrasse 9, 8092 Zurich, Switzerland, E-mail: sturlas@ethz.ch

This supplementary information includes:

- Supplementary tables 1 – 6
- Supplementary figures 1 – 5

Supplementary table 1. Summary of selected in vitro assays for liver steatosis

| Reference | Cells | Key events | Assay format | IVIVE | Chemical(s) tested |
| --- | --- | --- | --- | --- | --- |
| (Donato et al. 2012) | HepG2 | Lipid content  ROS generation  Mitochondrial membrane potential  Cell viability | Simultaneous assessment of key events | none | 22 known steatotic and nonsteatotic pharmaceuticals |
| (Tolosa et al. 2016) | HepaRG | Lipid content  Viability  Mitochondrial membrane potential  ROS production | Simultaneous assessment of key events | Comparison of in vitro concentration to Cmax values | 28 known steatotic and nonsteatotic pharmaceuticals |
| (Luckert et al. 2018) | HepaRG | Nuclear receptor activation  Gene and protein expression  Lipid accumulation  Mitochondrial respiration  Formation of fatty liver cells | Battery of assays of key events | none | cyproconazole |
| (Shah et al. 2021) | Rat hepatocytes | Endoplasmic reticulum stress  Mitochondrial function  Lysosomal mass  Steatosis  Apoptosis  DNA texture  Nuclear size  Cell number | Simultaneous assessment of key events | Prediction of administered equivalent doses using toxicokinetic modeling and comparing to rat in vivo exposure data | 51 chemicals known to induce hepatotoxicity |
| This study | HepaRG | Lipid accumulation  Mitochondrial membrane potential  Oxidative stress  Nuclear morphology | Simultaneous assessment of key events | Prediction of oral equivalent doses using PBPK modeling and reverse dosimetry. Comparison to human exposure data | 30 known steatotic, known non-steatotic, and unknown potential steatotic pharmaceutical and food-related chemicals |

IVIVE: in vitro to in vivo extrapolation

Supplementary table 2. Reference chemicals used to develop the high-content screening assay.

| Name | Structure | EC_50_ µM (95% CI)^a^ | Class / Use | Molecular targets | Reference |
| --- | --- | --- | --- | --- | --- |
| amiodarone | 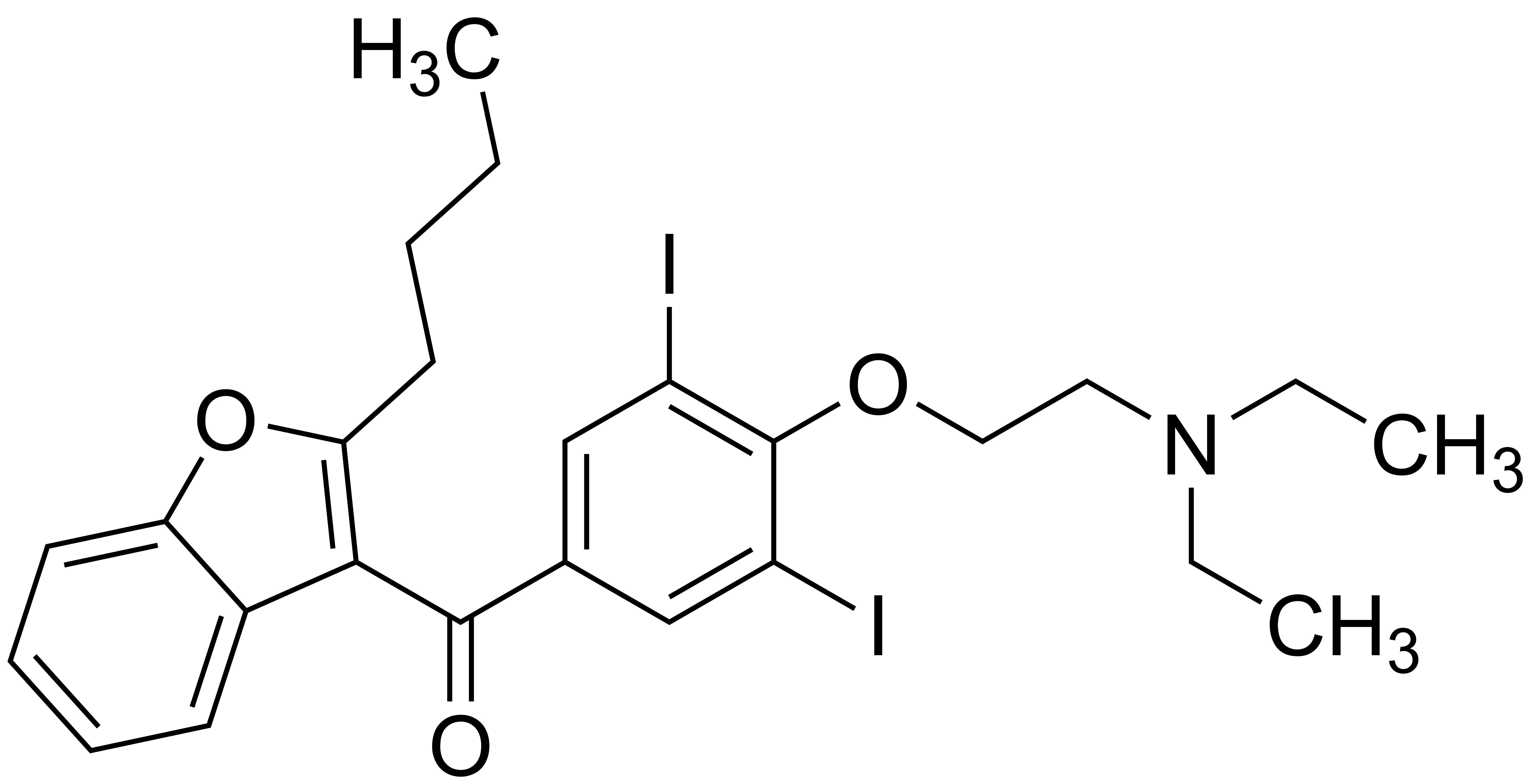 | 45.1  (41.2 – 48.8) | Anti-arrhythmic drug | Inhibition of phospholipase A  Inhibition of CPT-1  Inhibition of electron transport chain complexes I,II,III | (Anthérieu et al. 2011; Schumacher and Guo 2015) |
| caffeine | 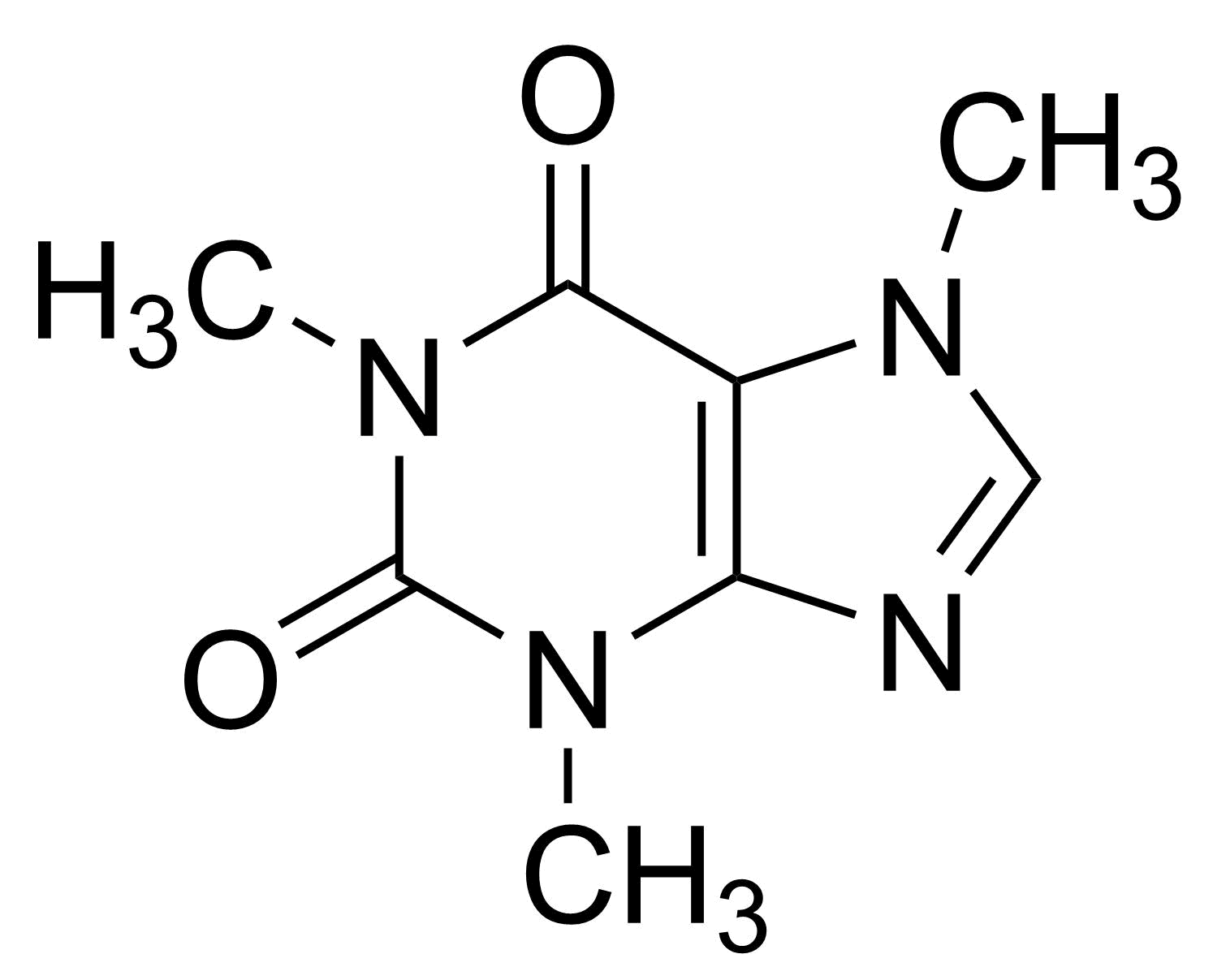 | 6526  (2125 – 23900) | Psychoactive drug | - | - |
| etomoxir | 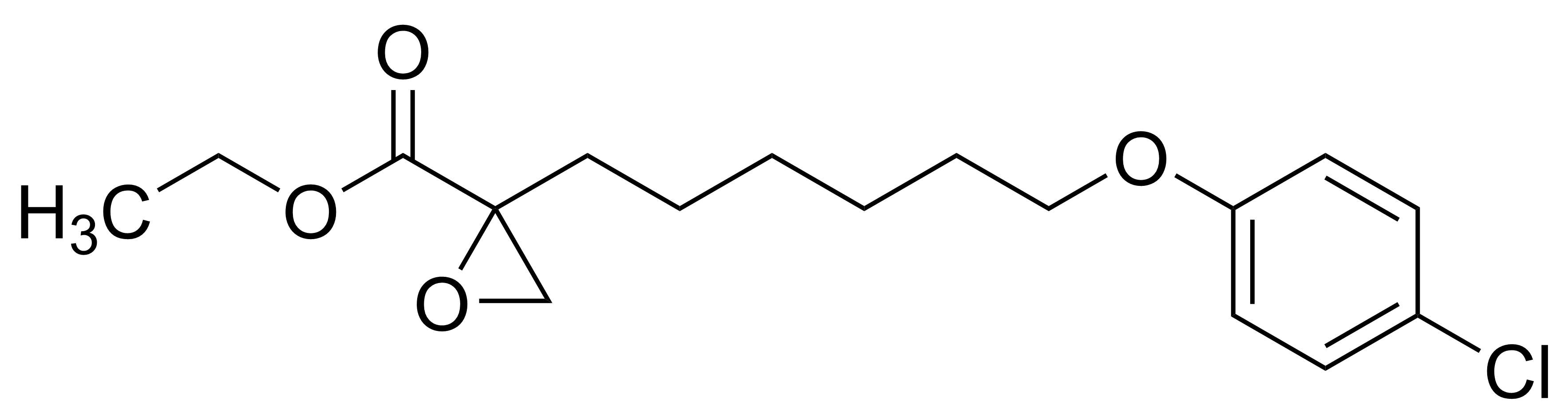 | 143.8  (121.8 – 213.4) | Anti-diabetic (type 2)  Treatment against heart failure | Irreversible CPT-1 inhibitior | (Merrill et al. 2002) |
| FCCP | 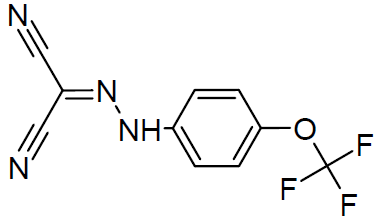 | NA | Lipophilic weak acid | Increases permeability of lipid membranes to protons leading to disruption of MMP | (Kane et al. 2018) |
| fialuridine | 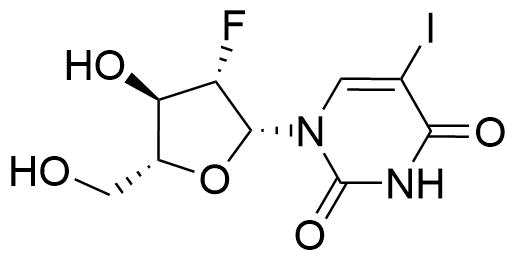 | NA | investigational nucleoside analogue for chronic hepatitis | Inhibition of mitochondrial DNA polymerase γ | (McKenzie et al. 1995; Lewis et al. 2003) |
| lomitapide | 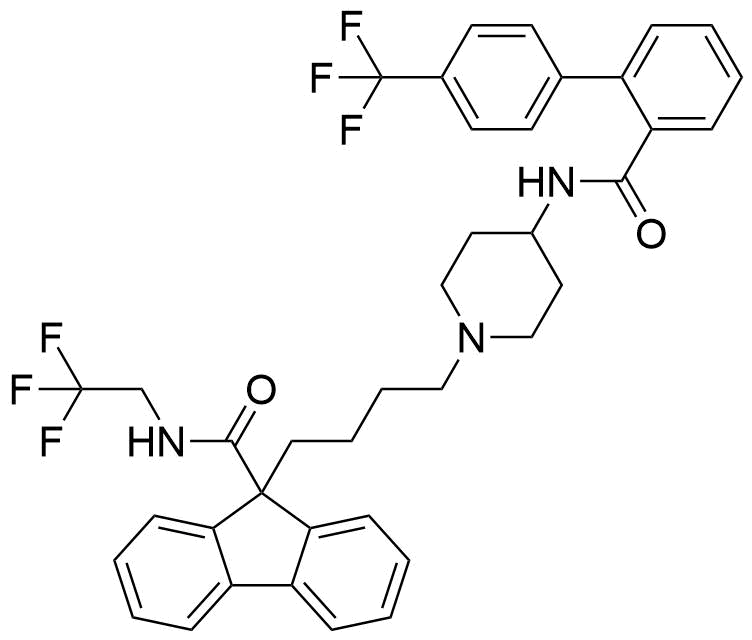 | 13.5  (12.4 – 17.3) | Anti-hypercholesterolemic | MTP inhibtion | (Lin et al. 2014) |
| menadione | 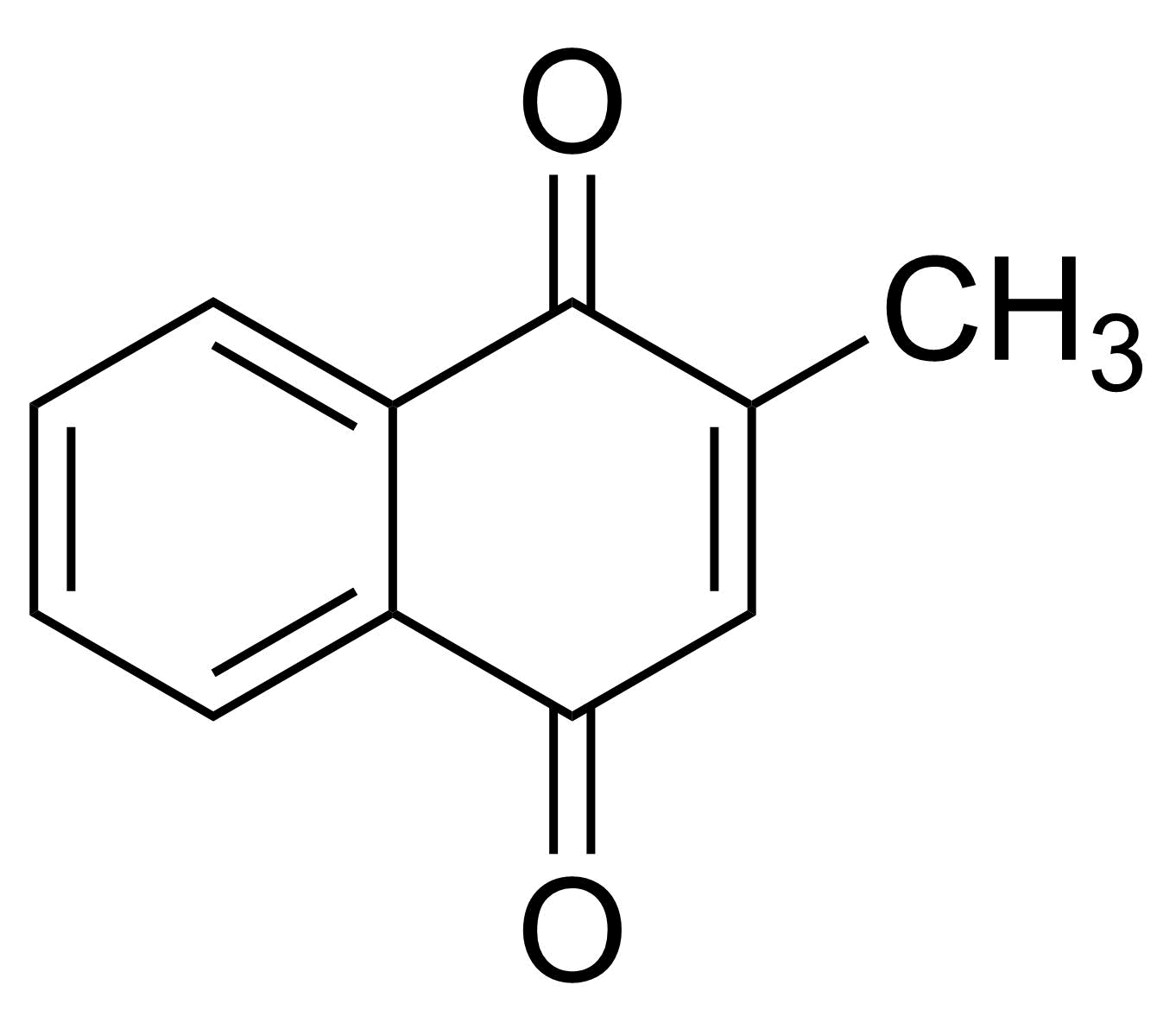 | 59.1  (50.4 – 71.7) | Synthetic Vitamin K | Upregulates P53  Increase of ROS | (Al-Suhaimi 2014) |
| metformin | 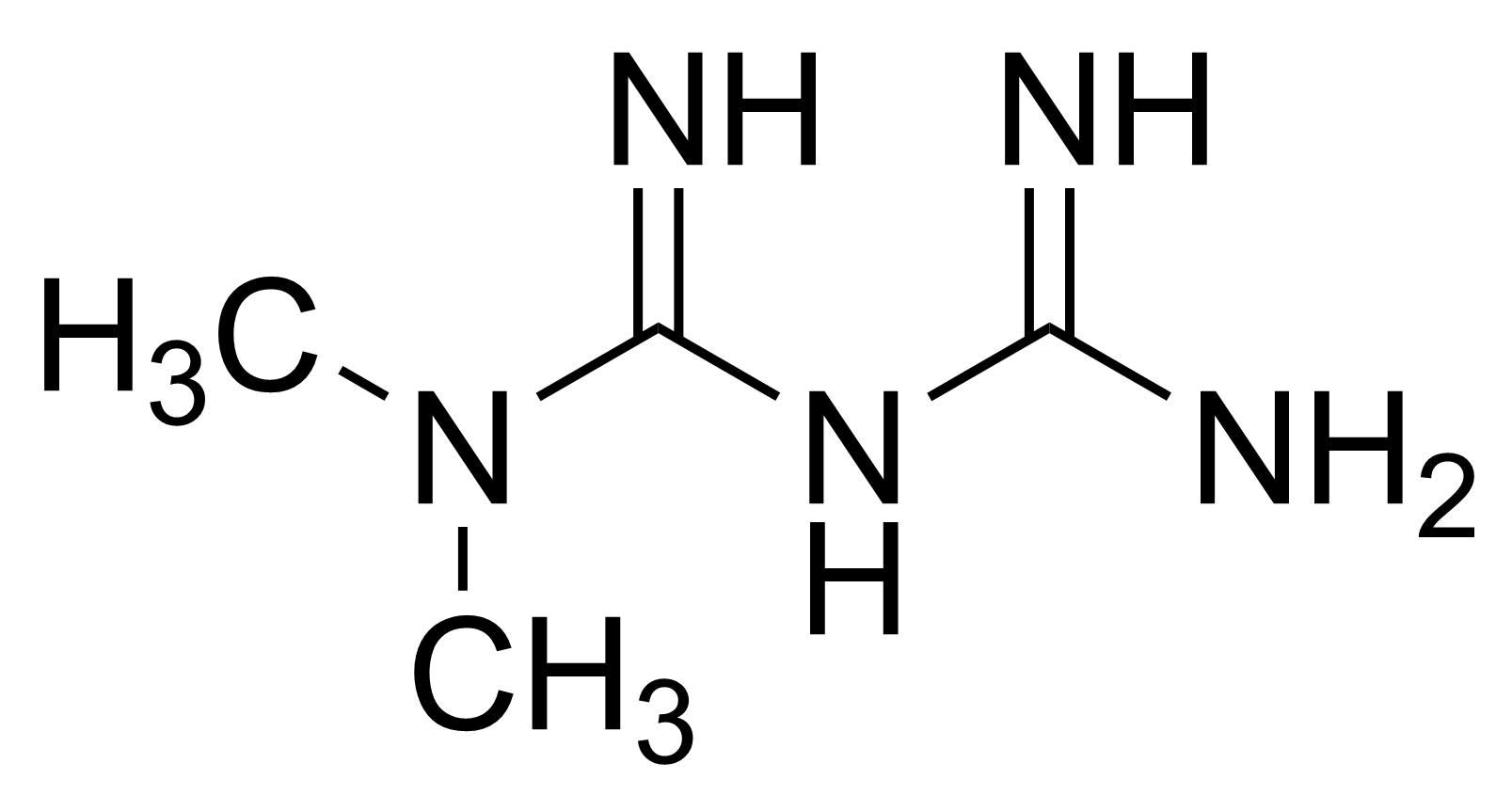 | 27’600  (10’000 – 105’500) | Anti-diabetic (type 2) | Inhibition of mitochondrial complex I | (Pernicova and Korbonits 2014) |
| oleic acid/palmitic acid | 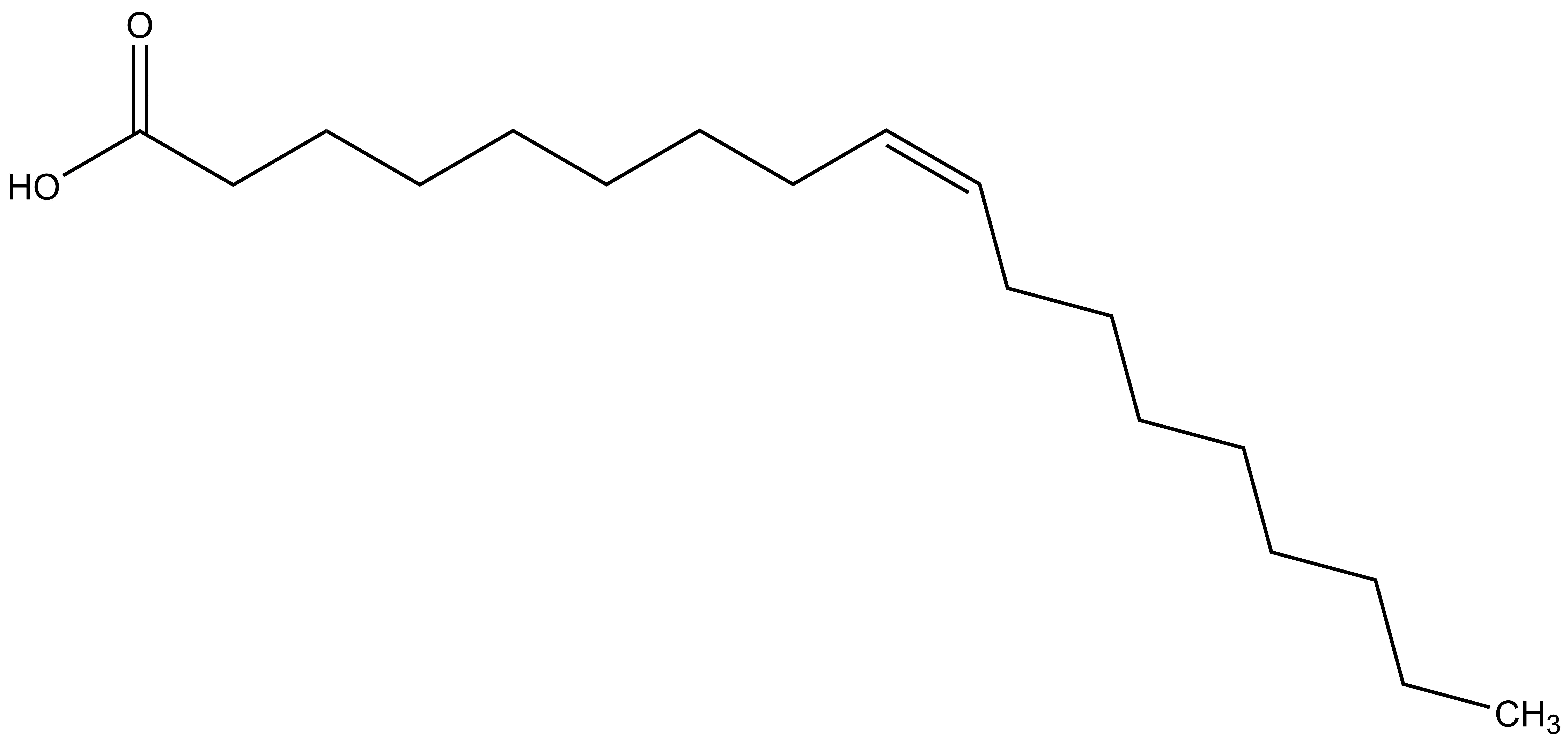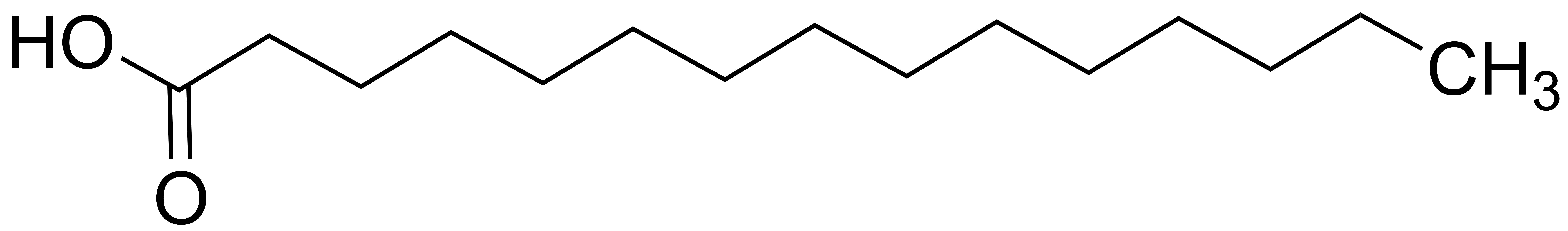 | NA | Dietary components | FATP  PPARγ | (Schaffer and Lodish 1995; Ricchi et al. 2009) |
| rotenone | 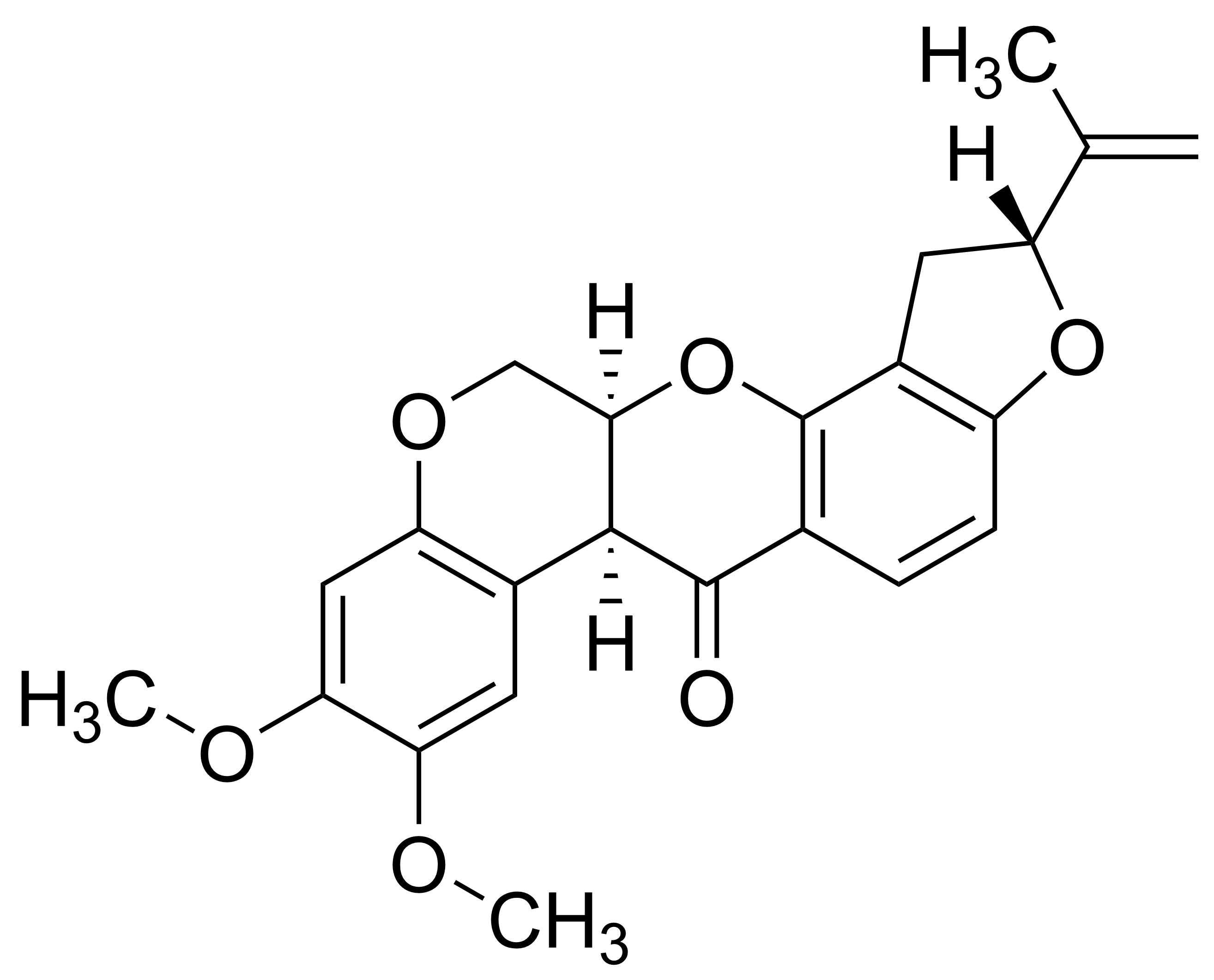 | 18.9  (10.8 – 71.8) | Insecticide | Selective mitochondrial complex I inhibitor | (Le et al. 2012; Heinz et al. 2017) |
| Sodium arsenite | 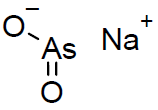 | NA | Metal | Increase in ROS by cycling between oxidation states | (Flora 2011) |
| T0901317 | 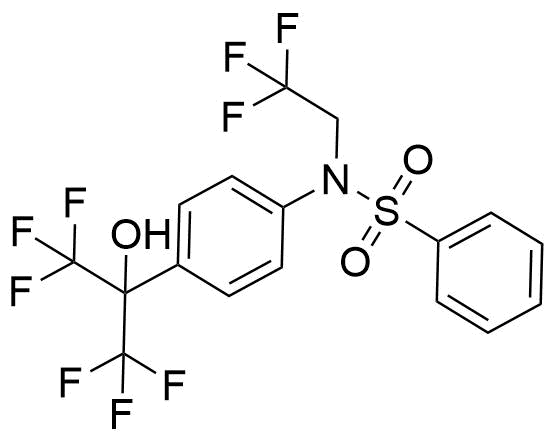 | 42.9  (41 – 46) | synthetic ligand | LXR and PXR agonist  Increase expression of SREBP-1c and ChREBP | (Mitro et al. 2007; Cha and Repa 2007) |
| tetracycline | 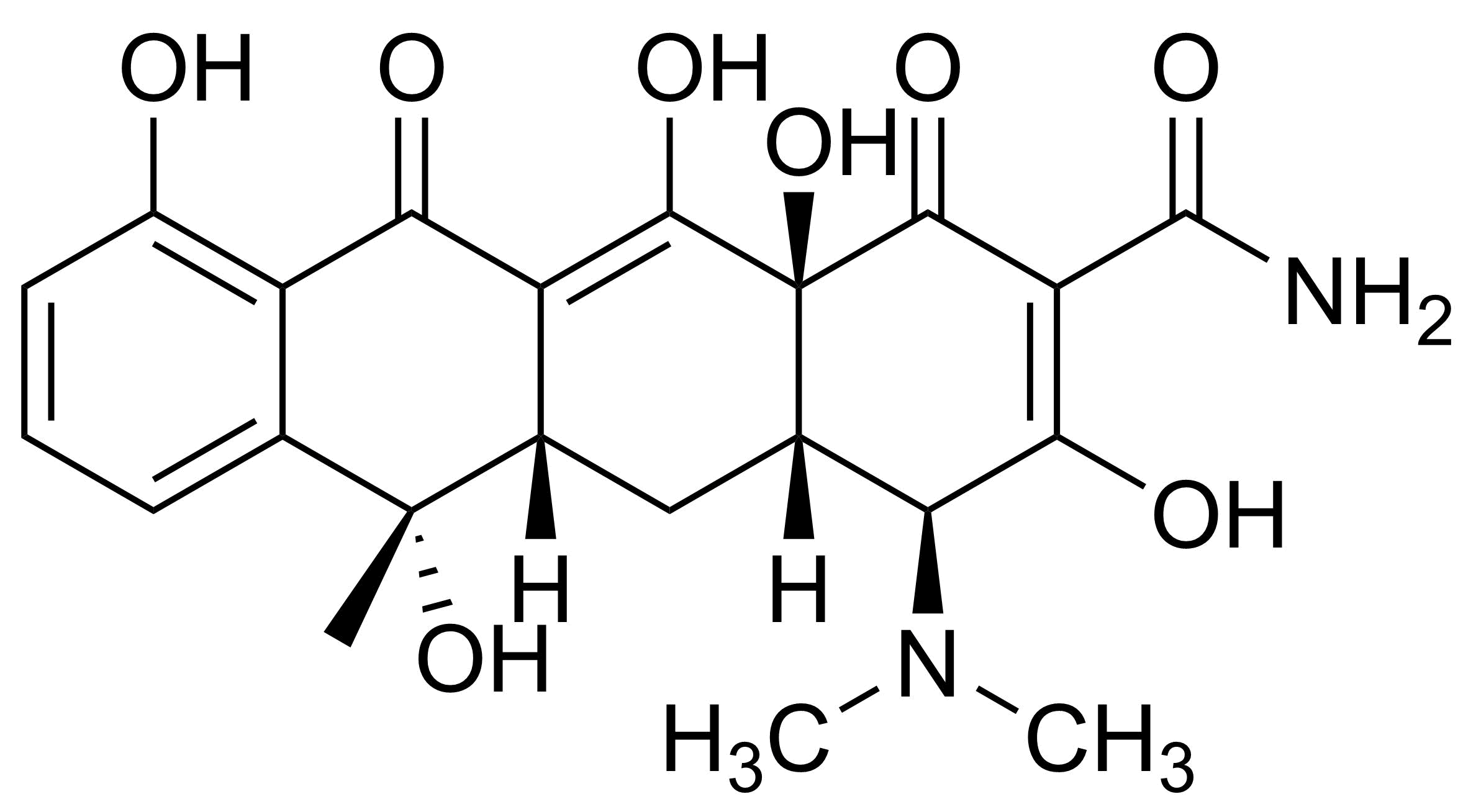 | 237.5  (201.6-373.4) | Antibiotic | Decrease in β-oxidation  Inhibition of MTP  Upregulation of PPARγ and SREBP1-c | (Anthérieu et al. 2011; Schumacher and Guo 2015) |
| valproic acid | 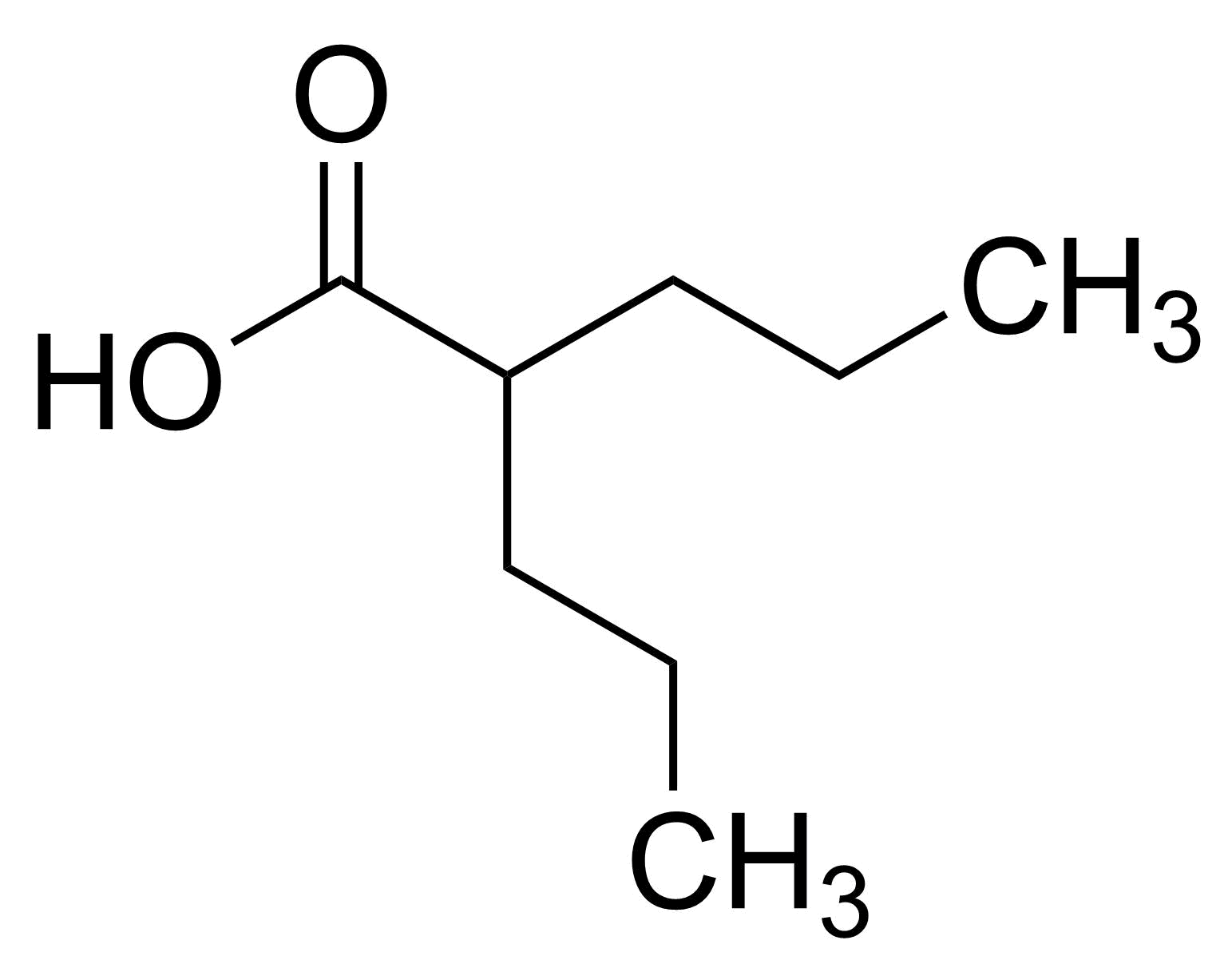 | 13’900  (5695 – 254’300)) | Anti-convulsant | Inhibition of β-oxidation  Upregulation of CD36  Upregulation of DGAT2 | (Schumacher and Guo 2015; Bai et al. 2017) |
| Wy14643 | 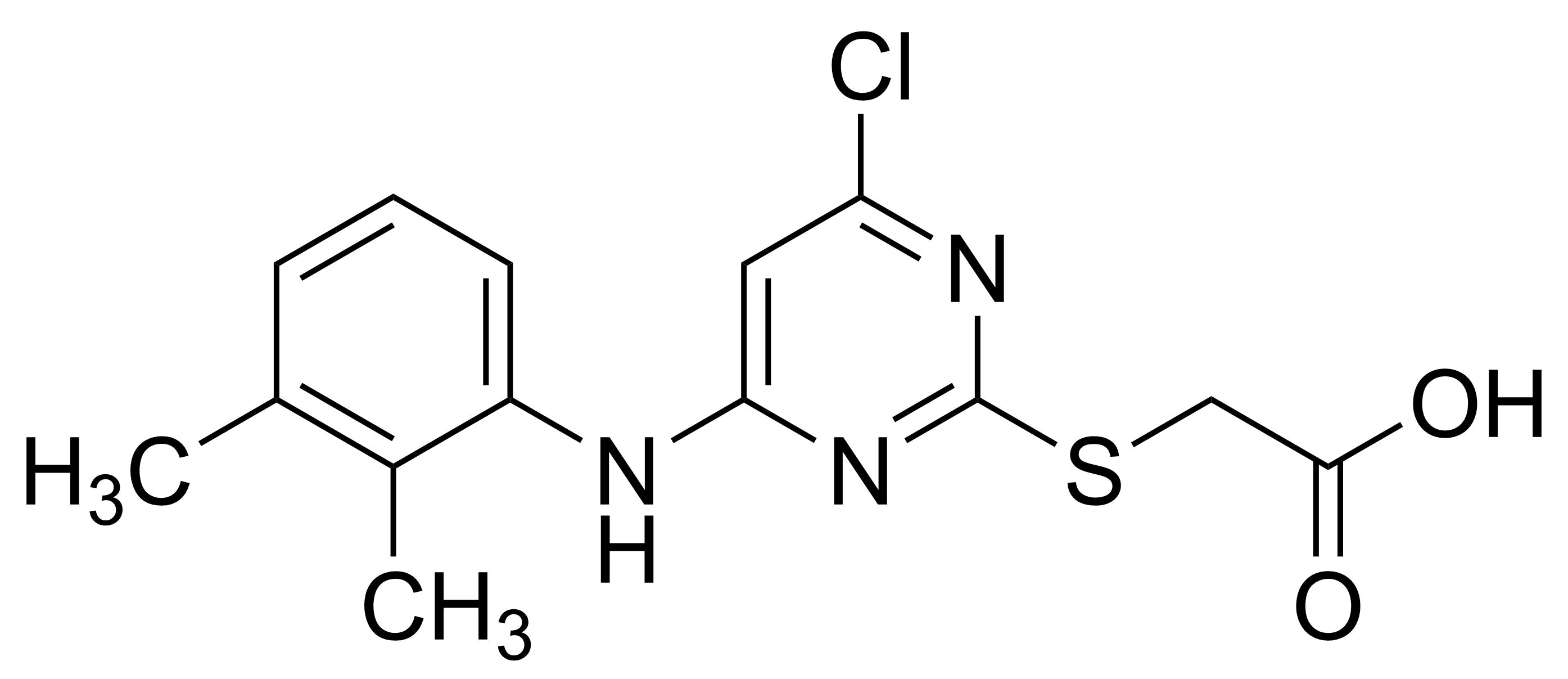 | NA | Experimental chemical to lower serum cholesterol | PPARα agonist  Increase in oxidative stress in mouse liver | (Woods et al. 2007) |
| β-naphthoflavone | 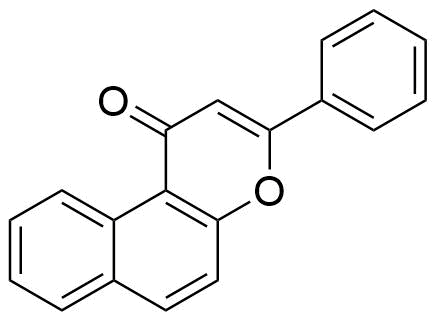 | NA | Model substance (polyaromatic hydrocarbon-like) | Aryl-hydrocarbon receptor agonist | (Lee et al. 2010; Jennings et al. 2014) |

^a^from cell viability (WST-1)

| Chemical | LA^1^ | MMP^1^ | OS^1^ | NM^1^ | Median Css^2^ (µM) | *In vivo* Css (µM) | Species | *In vivo* Css reference |
| --- | --- | --- | --- | --- | --- | --- | --- | --- |
| lomitapide | - | 0.58 | 10.5 | 4 | 0.0017 | 0.0002^3^ | human | (Aegerion Pharmaceuticals 2012) |
| fialuridine | - | 6.3 | - | 45.1 | 0.2 | 0.6^3^ | human | (Bowsher et al. 1994) |
| menadione | 2.8 | - | - | 8.1 | 5.7 | 12.6^4^ | rabbit | (Hu et al. 1996) |
| caffeine | - | - | - | - | 7.8 | 10.8 – 53.6 | human | (Birkett and Miners 1991) |
| WY-14643 | 40.98 | 38.1 | - | 386.3 | 66 | 2.5^3,5^ | rat | (Cunningham 2007) |
| metformin | 987.1 | 16’887 | - | 1936.5 | 9.6 | 5.2 | human | (Graham et al. 2011) |
| β-naphthoflavone | - | - | - | - | 32 | 2.3^3^ | rat | (Adedayo Adedoyin, Leon Arons 1993b) |
| amiodarone | 8.5 | 13.8 | 5.35 | 14.95 | 5.2 | 5.4 – 6.0 | human | (Freedman and Somberg 1991) |
| tetracycline | 32.9 | 218.43 | 69.7 | 32.7 | 12.6 | 9.7^3^ | human | (Welling et al. 1977) |
| valproic acid | 569.95 | 1311.3 | 1379.8 | 611.6 | 203.1 | 346.7 – 693.4 | human | (Vasudev et al. 2001) |

Supplementary table 3. Comparison of benchmark concentration and predicted Css with published pharmacokinetic values**.** Where indicated, in vivo derived Css values were calculated using following formula: Css = (Dose*F)/(Cl*dosing interval) or Css = k0/Cl with F = bioavailability, Cl = clearance, k0 = infusion rate and Css = steady state concentration.

LA: lipid accumulation, MMP: mitochondrial membrane potential, OS: oxidative stress, NM: nuclear morphology, ^1^Benchmark concentration (µM) per imaging endpoint calculated with BMDS software, ^2^Derived from IVIVE, ^3^calculated with formula described above,^4^in rabbits,^5^in rats


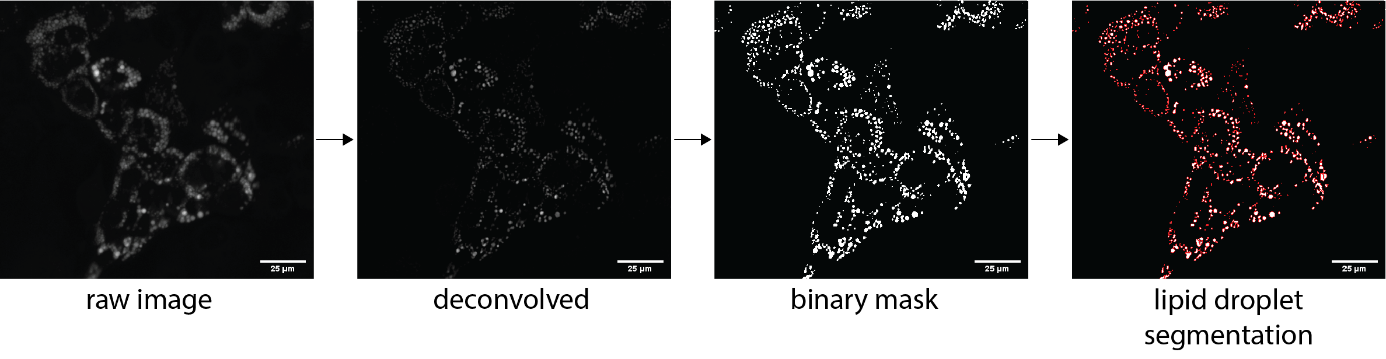


Supplementary figure 1. Lipid droplet quantification. Input was a raw fluorescent image stained with Bodipy 505/515 to identify lipid droplets. Then background was substracted and deconvolved using Fiji. These images were then loaded into CellProfiler and a binary mask was created. Based on this binary mask, single lipid droplets can be segmented. Scale bar = 25 µm


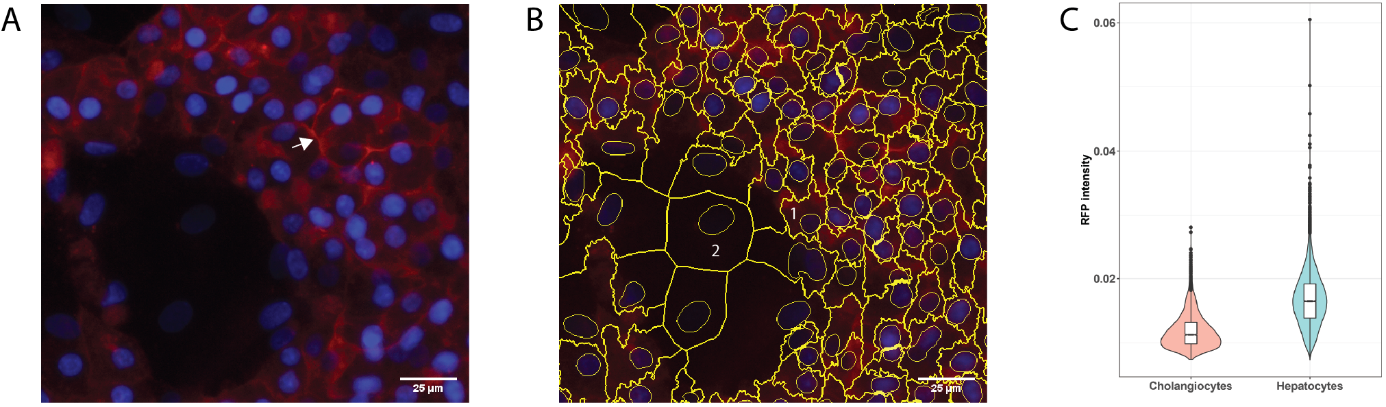


Supplementary figure 2. Anti-ASGPR1 staining in HepaRG cells. A) Representative fluorescent image of HepaRG cells stained ASGPR1 (hepatocytes; red) and Hoechst (nuclei; blue). Staining is observed in hepatocyte island whereas cholangiocytes remain unstained. Membrane localization of ASGPR1 was visible (white arrow). B) An example image of segmented cells (yellow lines). Hepatocytes were identified using two criteria: (i) a smaller cytoplasm compared to cholangiocytes (ii) stained positive for ASGPR1. C) Single cell quantification of ASGPR1 intensity in hepatocytes and cholangiocytes using single cell analysis pipeline. Between 5000 and 10’000 cells were analyzed per cell type. Scale bar = 25 µm

Supplementary table 4: Physicochemical properties and in vivo toxicokinetic data for selected chemicals where Clint was manually calculated using formula 3 and added to the httk database

| Name | CAS | MW (g/mol) | LogP | Fup | In vivo clearance (L/h) | Calculated Clint (ul/min/10^6 cells) | Species | Literature |
| --- | --- | --- | --- | --- | --- | --- | --- | --- |
| Lomitapide | 182431-12-5 | 693.7 | 8.2 | 0.002 | 573.4656 (after 50 mg p.o.) | 48.25141 | Human | (Aegerion Pharmaceuticals 2012) |
| Fialuridine | 69123-98-4 | 372.09 | -0.9 | 0.375 | Total clearance: 6.27  Renal clearance: 4.21  Nonrenal clearance: 2.06 | 0.1733285 | Human | (Bowsher et al. 1994) |
| Metformin | 657-24-9 | 129.16 | -2.6 | 0.99 | Total clearance: 79.1  Renal clearance: 31.5  Nonrenal clearance: 31.5 | 4.005065 | Human | (Scheen 1996) |
| Beta-Naphthoflavone | 6051-87-2 | 272.3 | 4.4 | 0.04 | 1.3 (plasma clearance) | 23.36091 | Rat | (Adedayo Adedoyin, Leon Arons 1993a) |
| Menadione | 58-27-5 | 172.18 | 2.2 | 0.2 | 49.32 | 58.33511 | Rabbit | (Hu et al. 1996) |

Fup: fraction unbound plasma, Clint: in vitro intrinsic hepatic clearance

Supplementary table 5: Z’ and strictly standardized mean difference (SSMD) comparison of positive controls

| OAPA, LD | | |  | Amiodarone, OS | | |
| --- | --- | --- | --- | --- | --- | --- |
| Plate Nr. | Z' | SSMD |  | Plate Nr. | Z' | SSMD |
| 1 | 0.61 | 10.72 |  | 1 | 0.19 | 4.25 |
| 2 | 0.7 | 13.87 |  | 2 | 0.22 | 4.15 |
| 3 | 0.66 | 12.11 |  | 3 | 0.26 | 4.63 |
| mean | 0.656667 | 12.23333 |  | mean | 0.223333 | 4.343333 |
|  |  |  |  |  |  |  |
|  |  |  |  |  |  |  |
| Rotenone, MD | | |  | **Menadione, NM** | | |
| Plate Nr. | Z' | SSMD |  | Plate Nr. | Z' | SSMD |
| 2 | 0.147 | -4.7 |  | 1 | 0.494 | 7.64 |
| 3 | 0.45 | -7.63 |  | 2 | -0.065 | 3.19 |
| 4 | 0.12 | -4.33 |  | 3 | 0.06 | 3.76 |
| mean | 0.239 | -5.55333 |  | mean | 0.163 | 4.863333 |


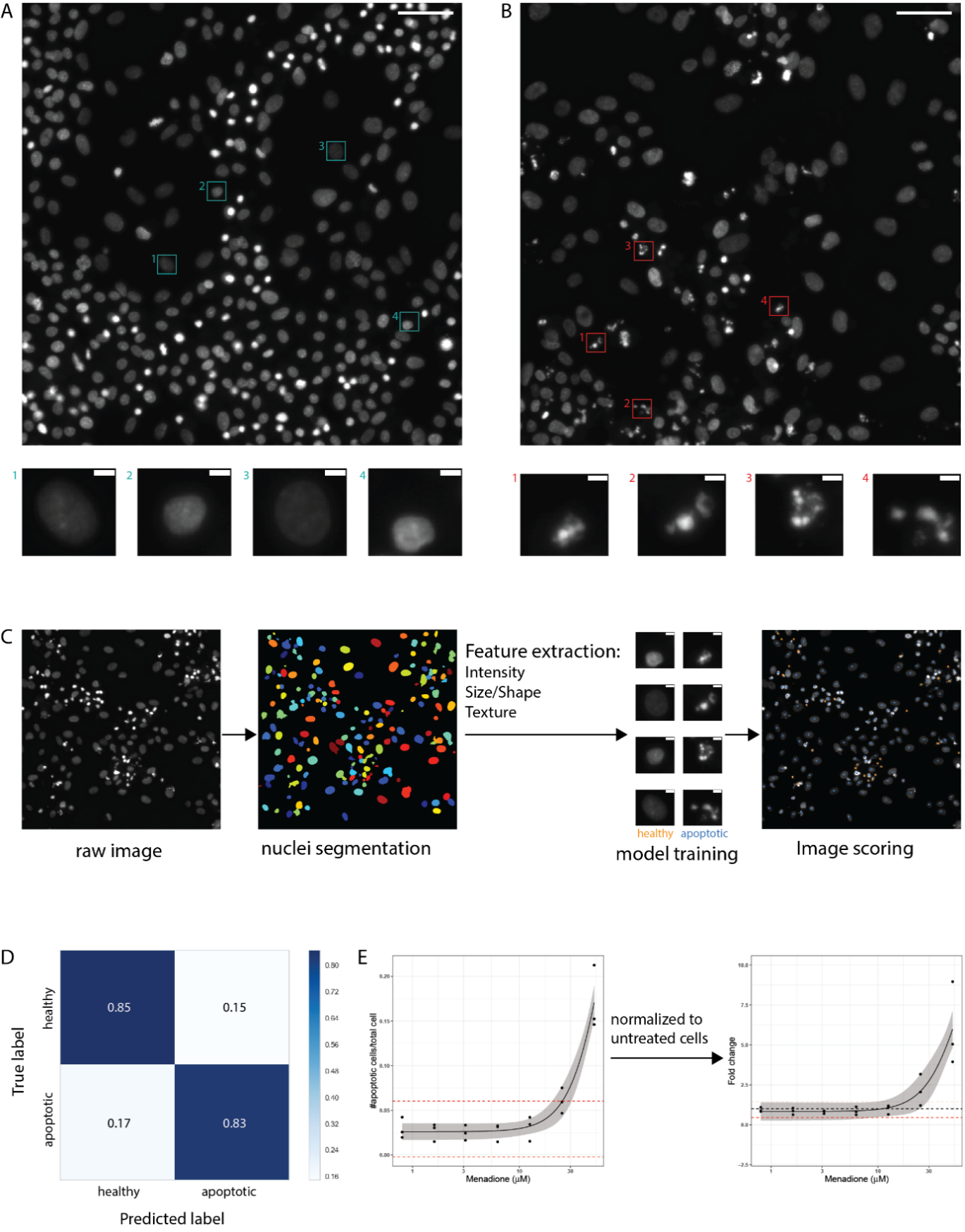


Supplementary figure 3.Machine learning approach to quantify number of apoptotic cells. A) Representative fluorescent image of untreated HepaRG cells stained with Hoechst. In small boxes are examples of healthy nuclei. Scale bar in big picture is 25 µm and in the small boxes 5 µm. B) Representative fluorescent image of HepaRG cells treated with 50 µM Menadione and stained against Hoechst. In small boxes are examples of apoptotic nuclei. Scale bar in big picture is 25 µm and in the small boxes 5 µm. C) Description of machine learning pipeline. First, a raw nuclei image is segmented and over 200 features (intensity, size/shape and texture) per nuclei were extracted. This features were then fed into a random forest classifier model to train the model. Subsequently, images were scored with the trained model. D) Confusion matrix of a scoring run. E) Normalization approach of apoptotic nuclei quantification. Number of apoptotic nuclei were counted and normalized to cell number. Then they were normalized to untreated cells to get fold change.

| **Name** | **Tested concentrations (µM)** | **EC_50_ (95% CI) in µM from cell viability assay** | **Use** | **Predicted activation of nuclear receptors** |
| --- | --- | --- | --- | --- |
| **Alpha-terpineol** | 125, 250, 500, 1000, 2000, 4000, 8000, 16000 (WST-1)  19.5, 39.1, 78.125, 156.25, 312.5, 625, 1250, 2500 (HCI) | 2565 (1853 – 3276) | Component of essential oils | none |
| **Deoxycholic acid** | 7.8125, 16.625, 31.25, 125, 250, 500, 1000 (WST-1)  2, 4, 8, 16.125, 33.25, 62.5, 125, 250 (HCI) | 246 (211 – 282) | Emulsifier and secondary bile acid | none |
| **Orotic acid** | 62.5, 125, 250, 500, 1000, 2000, 3000, 4000 (WST-1)  16.125, 31.25, 62.5, 125, 250, 500, 1000, 2000 (HCI) | NA | Mineral carrier in dietary supplements | THR |
| **Uric acid** | 7.8125, 16.625, 31.25, 125, 250, 500, 1000 (WST-1)  7.8125, 16.625, 31.25, 125, 250, 500, 1000 (HCI) | NA | Metabolite of purine nucleotides | None |
| **Tartrazine** | 16.625, 31.25, 125, 250, 500, 1000, 10000 (WST-1)  16.625, 31.25, 125, 250, 500, 1000, 10000 (HCI) | NA | Food dye | none |
| **Fructose** | 24, 98, 390, 1560, 6250, 25000, 100000, 400000 (WST-1)  39.1,39.1, 78.125, 156.25, 312.5, 625, 1250, 2500, 5000 (HCI) | 6.6 (1.1 – 156258) | Food component | none |
| **Bisphenol A** | 50, 100, 200, 225, 250, 400, 800, 1600 (WST-1)  1.6, 3.215, 6.25, 12.5, 25, 50, 100, 200 (HCI) | 237 (2.18 – 2.56) | Plasticizer and contact material | LXR, AHR, AR, ER, GR, PR, THR |
| **Carbosulfan** | 0.01, 0.1, 1, 10, 100, 500, 750, 1000 (WST-1)  0.01, 0.1, 1, 10, 100, 500, 750, 1000 (HCI) | NA | Insecticide | PPAR, AHR, AR, ER, GR, PR, THR, PXR |
| **Vinclozolin** | 0.00001, 0.0001, 0.001, 0.01, 0.1, 1, 10, 100 (WST-1)  0.00001, 0.0001, 0.001, 0.01, 0.1, 1, 10, 100 (HCI) | NA | Fungicide | FXR, AHR, AR, ER, GR, PR, THR |
| **Mepanipyrim** | 0.01, 0.1, 1, 10, 50, 100, 150, 300 (WST-1)  0.01, 0.1, 1, 10, 50, 100, 150, 300 (HCI) | NA | Fungicide | AHR, AR, ER, GR, PR, THR |
| **Atrazine** | 1.15, 2.3, 4.6875, 9.375, 18.75, 37.5, 75, 150 (WST-1)  1.15, 2.3, 4.6875, 9.375, 18.75, 37.5, 75, 150 (HCI) | NA | Herbicide | none |
| **Carbofuran** | 0.33, 1, 3, 9, 27, 90, 300, 1000 (WST-1)  7.8125, 16.625, 31.25, 125, 250, 500, 1000 (HCI) | NA | Insecticide | none |
| **Fipronil** | 0.512, 1.280, 3.2, 8, 20, 50, 125, 500 (WST-1)  0.225, 0.45, 0.9, 1.875, 3.75, 7.5, 15, 30 (HCI) | 21 (15 – 27) | Insecticide | PPAR, AHR, AR, ER, GR, PR, THR, PXR |
| **Metazachlor** | 0.33, 1, 3, 9, 27, 90, 300, 1000 (WST-1)  1.175, 2.35, 4.6875, 9.375, 18.75, 37.5, 75, 150 (HCI) | 183 (106 – 261) | Herbicide | none |

Supplementary table 6: Food-related chemicals and pesticides selected for the application screen


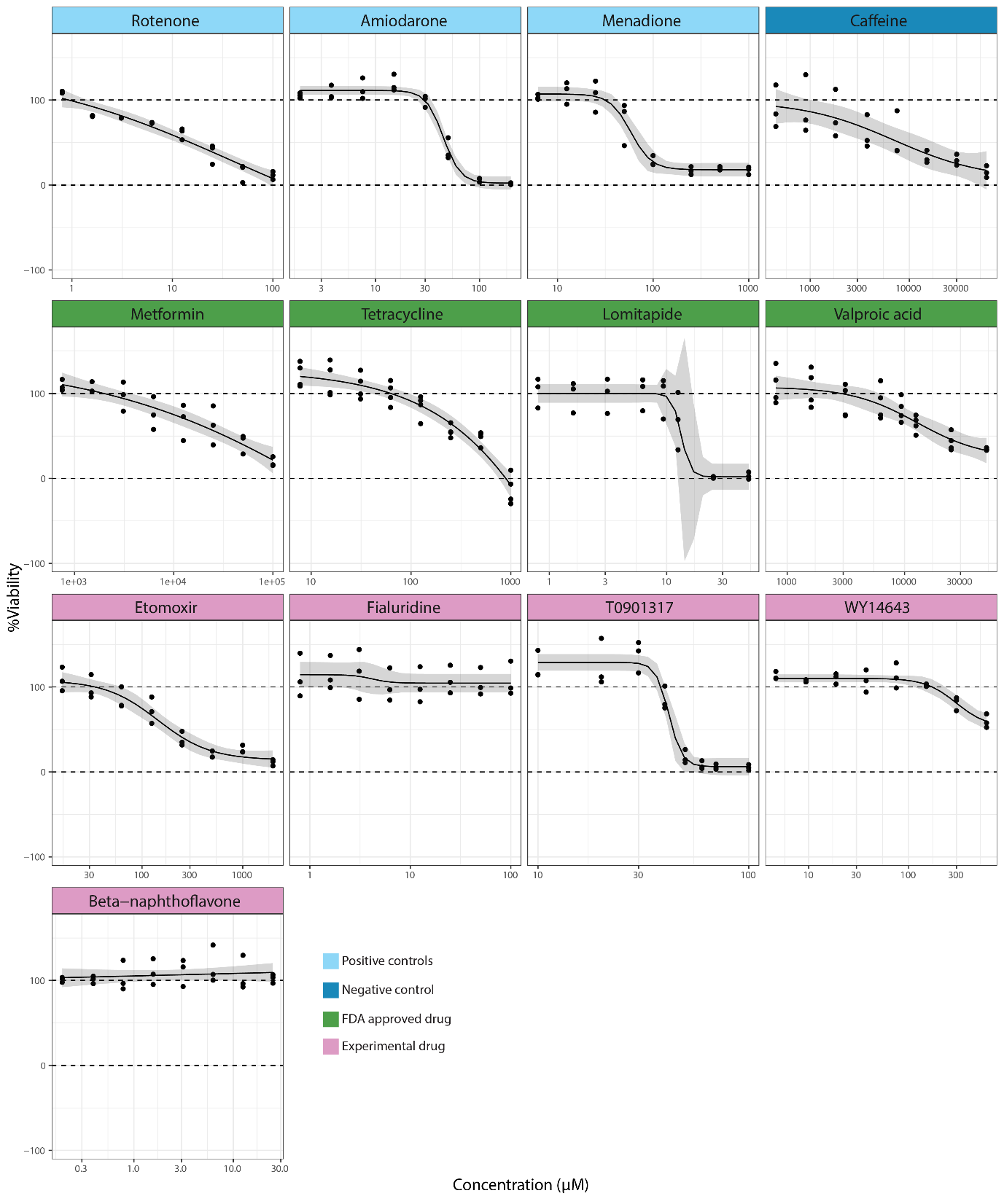


Supplementary figure 4. Cell viability of reference chemicals assessed by WST-1 assay. Chemicals were grouped based on their usage as controls or current approval. Grey area around non-linear regression represents the 95% CI. Black dotted line represents a fold change of 1 (DMSO control). All data were first normalized to total cell number and then to untreated cells. At least 3 independent experiments with 6 technical replicates per concentration per experiment were conducted for all chemicals


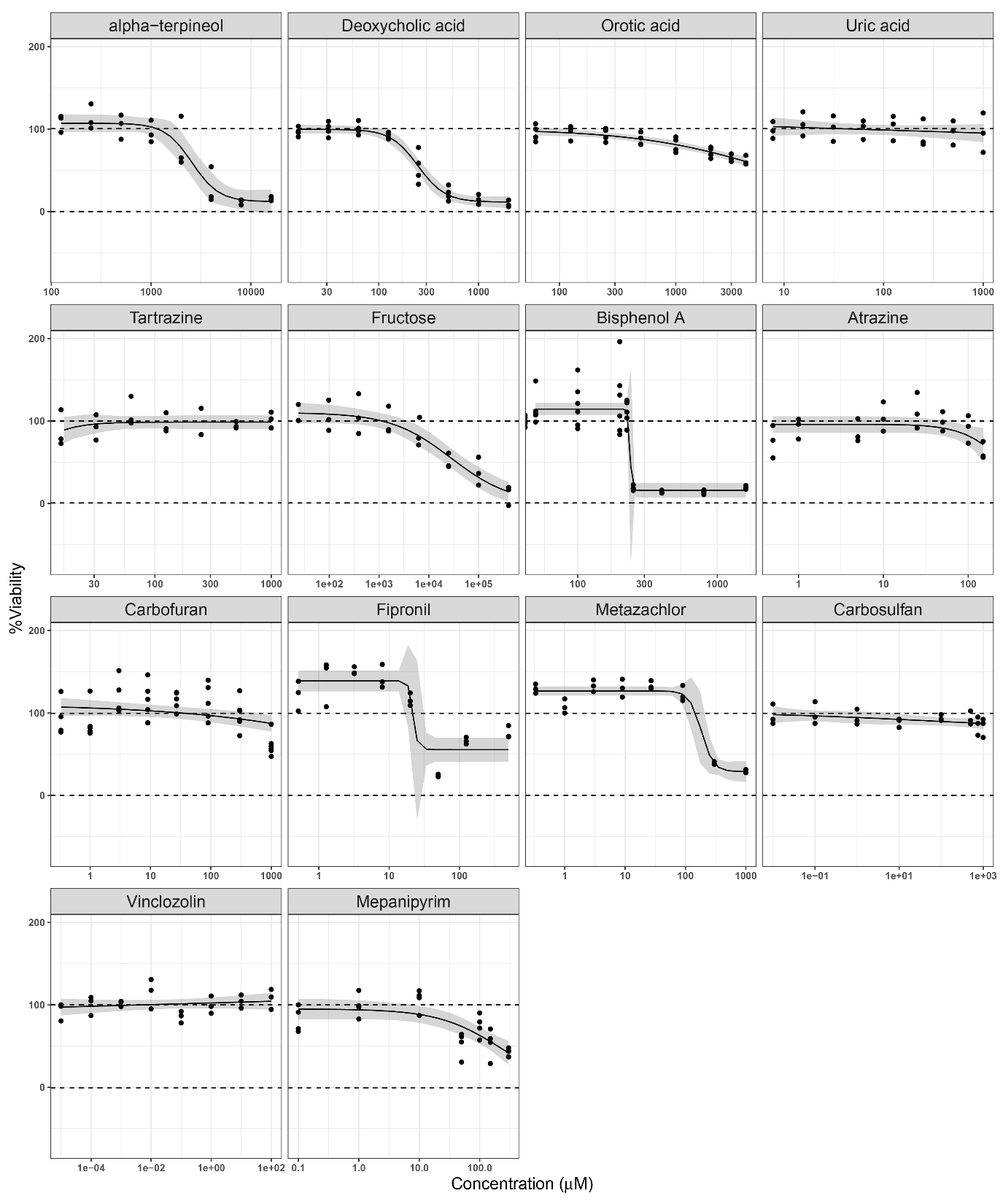


Supplementary figure 5. Cell viability of food-related chemicals assessed by WST-1 assay. Grey area around non-linear regression represents the 95% CI. Black dotted line represents a fold change of 1 (DMSO control). All data were first normalized to total cell number and then to untreated cells. At least 3 independent experiments with 6 technical replicates per concentration per experiment were conducted for all chemicals

**References**

Adedayo Adedoyin, Leon Arons JBH (1993a) Time-dependent disposition of b-naphthoflavone in the rat. Pharmacol Res 10:35–43

Adedayo Adedoyin, Leon Arons JBH (1993b) Time-dependent disposition of b-naphthoflavone in the rat. Pharmacol Res 10:35–43

Aegerion Pharmaceuticals (2012) Pharmacology/Toxicology review of NDA 203-858. In: Food and Drug Administration. https://www.accessdata.fda.gov/drugsatfda_docs/nda/2012/203858Orig1s000ClinpharmR.pdf

Al-Suhaimi E (2014) Molecular mechanisms of leptin and pro-apoptotic signals induced by menadione in HepG2 cells. Saudi J Biol Sci 21:582–588. https://doi.org/10.1016/J.SJBS.2014.03.002

Anthérieu S, Rogue A, Fromenty B, et al (2011) Induction of vesicular steatosis by amiodarone and tetracycline is associated with up-regulation of lipogenic genes in heparg cells. Hepatology 53:1895–1905. https://doi.org/10.1002/hep.24290

Bai X, Hong W, Cai P, et al (2017) Valproate induced hepatic steatosis by enhanced fatty acid uptake and triglyceride synthesis. Toxicol Appl Pharmacol 324:12–25. https://doi.org/10.1016/j.taap.2017.03.022

Birkett D, Miners J (1991) Caffeine renal clearance and urine caffeine concentrations during steady state dosing. Implications for monitoring caffeine intake during sports events. Br J Clin Pharmacol 31:405–408. https://doi.org/10.1111/j.1365-2125.1991.tb05553.x

Bowsher RR, Compton JA, Kirkwood JA, et al (1994) Sensitive and Specific Radioimmunoassay for Fialuridine - Initial Assessment of Pharmacokinetics After Single Oral Doses To Healthy-Volunteers. Antimicrob Agents Chemother 38:2134–2142

Cha JY, Repa JJ (2007) The Liver X Receptor (LXR) and hepatic lipogenesis: The carbohydrate-response element-binding protein is a target gene of LXR. Journal of Biological Chemistry 282:743–751. https://doi.org/10.1074/jbc.M605023200

Cunningham ML (2007) NTP technical report on the toxicity studies of Wy-14643

Donato MT, Tolosa L, Jiménez N, et al (2012) High-Content Imaging Technology for the Evaluation of Drug-Induced Steatosis Using a Multiparametric Cell-Based Assay. J Biomol Screen 17:394–400. https://doi.org/10.1177/1087057111427586

Flora SJS (2011) Arsenic-induced oxidative stress and its reversibility. Free Radic Biol Med 51:257–281. https://doi.org/10.1016/J.FREERADBIOMED.2011.04.008

Freedman MD, Somberg JC (1991) Pharmacology and Pharmacokinetics of Amiodarone. The Journal of Clinical Pharmacology 31:1061–1069. https://doi.org/10.1002/j.1552-4604.1991.tb03673.x

Graham GG, Punt J, Arora M, et al (2011) Clinical Pharmacokinetics of Metformin. Clin Pharmacokinet 50:81–98. https://doi.org/10.2165/11534750-000000000-00000

Heinz S, Freyberger A, Lawrenz B, et al (2017) Mechanistic Investigations of the Mitochondrial Complex I Inhibitor Rotenone in the Context of Pharmacological and Safety Evaluation. Sci Rep 7:45465. https://doi.org/10.1038/srep45465

Hu OY-P, Wu C-Y, Chan W-K, et al (1996) A pharmacokinetic study with the high-dose anticancer agent menadione in rabbits. Biopharm Drug Dispos 17:493–499. https://doi.org/10.1002/(SICI)1099-081X(199608)17:6<493::AID-BDD972>3.0.CO;2-6

Jennings P, Schwarz M, Landesmann B, et al (2014) SEURAT-1 liver gold reference compounds: a mechanism-based review. Arch Toxicol 88:2099–2133. https://doi.org/10.1007/s00204-014-1410-8

Kane MS, Paris A, Codron P, et al (2018) Current mechanistic insights into the CCCP-induced cell survival response. Biochem Pharmacol 148:100–110. https://doi.org/10.1016/J.BCP.2017.12.018

Le TT, Ziemba A, Urasaki Y, et al (2012) Label-free evaluation of hepatic microvesicular steatosis with multimodal coherent anti-Stokes Raman scattering microscopy. PLoS One 7:e51092. https://doi.org/10.1371/journal.pone.0051092

Lee JH, Wada T, Febbraio M, et al (2010) A novel role for the dioxin receptor in fatty acid metabolism and hepatic steatosis. Gastroenterology 139:653–663. https://doi.org/10.1053/j.gastro.2010.03.033

Lewis W, Day BJ, Copeland WC (2003) Mitochondrial toxicity of NRTI antiviral drugs: An integrated cellular perspective. Nat Rev Drug Discov 2:812–822

Lin M, Zhao S, Shen L, Xu D (2014) Potential approaches to ameliorate hepatic fat accumulation seen with MTP inhibition. Drug Saf 37:213–224

Luckert C, Braeuning A, de Sousa G, et al (2018) Adverse Outcome Pathway-Driven Analysis of Liver Steatosis *in Vitro* : A Case Study with Cyproconazole. Chem Res Toxicol 31:784–798. https://doi.org/10.1021/acs.chemrestox.8b00112

McKenzie R, Fried MW, Sallie R, et al (1995) Hepatic Failure and Lactic Acidosis Due to Fialuridine (FIAU), an Investigational Nucleoside Analogue for Chronic Hepatitis B. New England Journal of Medicine 333:1099–1105. https://doi.org/10.1056/NEJM199510263331702

Merrill CL, Ni H, Yoon LW, et al (2002) Etomoxir-induced oxidative stress in HepG2 cells detected by differential gene expression is confirmed biochemically. Toxicological Sciences 68:93–101. https://doi.org/10.1093/toxsci/68.1.93

Mitro N, Vargas L, Romeo R, et al (2007) T0901317 is a potent PXR ligand: Implications for the biology ascribed to LXR. FEBS Lett 581:1721–1726. https://doi.org/10.1016/j.febslet.2007.03.047

Pernicova I, Korbonits M (2014) Metformin-Mode of action and clinical implications for diabetes and cancer. Nat Rev Endocrinol 10:143–156

Ricchi M, Odoardi MR, Carulli L, et al (2009) Differential effect of oleic and palmitic acid on lipid accumulation and apoptosis in cultured hepatocytes. J Gastroenterol Hepatol 24:830–840. https://doi.org/10.1111/j.1440-1746.2008.05733.x

Schaffer JE, Lodish HF (1995) Molecular Mechanism of Long-Chain Fatty Acid Uptake. Trends Cardiovasc Med 5:218–224

Scheen AJ (1996) Clinical Pharmacokinetics of Metformin. Clin Pharmacokinet 30:359–371

Schumacher JD, Guo GL (2015) Mechanistic review of drug-induced steatohepatitis. Toxicol Appl Pharmacol 289:40–7. https://doi.org/10.1016/j.taap.2015.08.022

Shah I, Antonijevic T, Chambers B, et al (2021) Estimating Hepatotoxic Doses Using High-Content Imaging in Primary Hepatocytes. Toxicological Sciences. https://doi.org/10.1093/TOXSCI/KFAB091

Tolosa L, Gómez-Lechón MJ, Jiménez N, et al (2016) Advantageous use of HepaRG cells for the screening and mechanistic study of drug-induced steatosis. Toxicol Appl Pharmacol 302:1–9. https://doi.org/10.1016/j.taap.2016.04.007

Vasudev K, Das S, Goswami U, Tayal G (2001) Pharmacokinetics of valproic acid in patients with bipolar disorder. Journal of Psychopharmacology 15:187–190. https://doi.org/10.1177/026988110101500305

Welling PG, Huang H, Koch PA, et al (1977) Bioavailability of tetracycline and doxycycline in fasted and nonfasted subjects. J Pharm Sci 66:549–552. https://doi.org/10.1002/jps.2600660423

Woods CG, Burns AM, Bradford BU, et al (2007) WY-14,643-induced cell proliferation and oxidative stress in mouse liver are independent of NADPH oxidase. Toxicological Sciences 98:366–374. https://doi.org/10.1093/toxsci/kfm104
